# Results of a multicentre randomised controlled trial of cochlear-sparing intensity-modulated radiotherapy versus conventional radiotherapy in patients with parotid cancer (COSTAR; CRUK/08/004)

**DOI:** 10.1016/j.ejca.2018.08.006

**Published:** 2018-11

**Authors:** Christopher M. Nutting, James P. Morden, Matthew Beasley, Shreerang Bhide, Audrey Cook, Emma De Winton, Marie Emson, Mererid Evans, Lydia Fresco, Simon Gollins, Dorothy Gujral, Kevin Harrington, Mano Joseph, Catherine Lemon, Linda Luxon, Qurrat van den Blink, Ruheena Mendes, Aisha Miah, Kate Newbold, Robin Prestwich, Martin Robinson, Paul Sanghera, Joanna Simpson, Muthiah Sivaramalingam, Narayanan Nair Srihari, Mark Sydenham, Emma Wells, Stephanie Witts, Emma Hall, the COSTAR Investigators

**Affiliations:** a Head and Neck Unit, Royal Marsden Hospital, London, United Kingdom; b The Institute of Cancer Research Clinical Trials and Statistics Unit, The Institute of Cancer Research, London, United Kingdom; c Bristol Cancer Institute, Bristol Haematology and Oncology Centre, United Kingdom; d Gloucestershire Oncology Centre, Cheltenham General Hospital, United Kingdom; e Department of Oncology, Royal United Hospital, Bath, United Kingdom; f Clinical Oncology, Velindre NHS Trust, United Kingdom; g Department of Oncology, University Hospitals of Coventry and Warwickshire, United Kingdom; h Department of Oncology, Glan Clwyd Hospital, Rhyl, United Kingdom; i Head and Neck Department, Imperial College Healthcare NHS Trust, United Kingdom; j Oncology and Haematology Directorate, The Royal Wolverhampton NHS Trust, United Kingdom; k Department of Oncology, Mount Vernon Hospital, United Kingdom; l University College London Ear Institute, United Kingdom; m Department of Oncology, Northampton General Hospital, United Kingdom; n Department of Oncology, University College Hospital, London, United Kingdom; o Sarcoma Unit, Royal Marsden Hospital, Sutton, United Kingdom; p Department of Clinical Oncology, St James's University Hospital, Leeds, United Kingdom; q Department of Clinical Oncology, Weston Park Hospital, Sheffield, United Kingdom; r Department of Oncology, University Hospitals Birmingham NHS Foundation Trust, United Kingdom; s Department of Oncology, Royal Sussex County Hospital, United Kingdom; t Department of Oncology, Royal Preston Hospital, United Kingdom; u Department of Oncology, Royal Shrewsbury Hospital, United Kingdom; v QA Physics, Royal Marsden Hospital, London, United Kingdom

**Keywords:** Radiotherapy, Head and neck cancer, Cochlear-sparing, IMRT, Hearing loss

## Abstract

**Purpose:**

About 40–60% of patients treated with post-operative radiotherapy for parotid cancer experience ipsilateral sensorineural hearing loss. Intensity-modulated radiotherapy (IMRT) can reduce radiation dose to the cochlea. COSTAR, a phase III trial, investigated the role of cochlear-sparing IMRT (CS-IMRT) in reducing hearing loss.

**Methods:**

Patients (pT1-4 N0-3 M0) were randomly assigned (1:1) to 3-dimensional conformal radiotherapy (3DCRT) or CS-IMRT by minimisation, balancing for centre and radiation dose of 60Gy or 65Gy in 30 daily fractions. The primary end-point was proportion of patients with sensorineural hearing loss in the ipsilateral cochlea of ≥10 dB bone conduction at 4000 Hz 12 months after radiotherapy compared using Fisher's exact test. Secondary end-points included hearing loss at 6 and 24 months, balance assessment, acute and late toxicity, patient-reported quality of life, time to recurrence and survival.

**Results:**

From Aug 2008 to Feb 2013, 110 patients (54 3DCRT; 56 CS-IMRT) were enrolled from 22 UK centres. Median doses to the ipsilateral cochlea were 3DCRT: 56.2Gy and CS-IMRT: 35.7Gy (p < 0.0001). 67/110 (61%) patients were evaluable for the primary end-point; main reasons for non-evaluability were non-attendance at follow-up or incomplete audiology assessment. At 12 months, 14/36 (39%) 3DCRT and 11/31 (36%) CS-IMRT patients had ≥10 dB loss (p = 0.81). No statistically significant differences were observed in hearing loss at 6 or 24 months or in other secondary end-points including patient-reported hearing outcomes.

**Conclusion:**

CS-IMRT reduced the radiation dose below the accepted tolerance of the cochlea, but this did not lead to a reduction in the proportion of patients with clinically relevant hearing loss.

## Highlights

• Ipsilateral hearing loss is experienced by 35–40% of patients having radiotherapy to their parotid.
• Reducing the radiation dose to the cochlea does not spare hearing loss.
• The radiosensitivity of the cochlea may be much greater than previously understood.

## 1 Introduction

Malignant parotid gland tumours represent 3–6% of head and neck cancers. Surgery is the mainstay of treatment [1]. Local recurrences occur in 20–70% of patients [1], [2], [3], [4]. Adjuvant, post-operative radiotherapy of 60–65Gy in 30 fractions given over 6 weeks is recommended for patients with high risk of recurrence [5], [6].

The ipsilateral cochlea is usually very close to the planning target volume (PTV) and often receives a dose greater than 50Gy [7] with conventional 3-dimensional conformal radiotherapy (3DCRT) techniques. As a consequence, clinically significant high-tone, sensorineural hearing loss (>10 dB) has been described in 40–60% of patients after radiotherapy [8], [9], [10], [11], [12], [13], [14], peaking at a frequency around 4000 Hz.

Intensity-modulated radiotherapy (IMRT) produces highly conformal radiation dose distributions. Cochlear-sparing IMRT (CS-IMRT) can reduce the dose to the ipsilateral cochlea, compared with 3DCRT, to below its accepted tolerance dose of 40–45Gy [7], [15]. COSTAR aimed to investigate whether CS-IMRT reduces sensorineural hearing loss.

## 2 Methods

### 2.1 Study design and participants

COSTAR is a phase III, parallel group, randomised controlled trial. Patients aged ≥18 years, WHO performance status 0–1 with histologically confirmed malignant primary parotid tumours (pT1-4, N0-3, M0) requiring post-operative adjuvant radiotherapy, were eligible. Exclusion criteria included previous head and neck radiotherapy, pre-existing severe hearing loss (hearing level of >60 dB in bone conduction threshold at 4000 Hz in ipsilateral cochlea) and need for chemotherapy. Patients were staged by diagnostic computed tomography (CT) or magnetic resonance imaging of head and neck and chest X-ray or CT of thorax. Resection status was documented from histopathology as R0 (resection margin >5 mm), R1 (1–5 mm) or R2 (<1 mm). Patients were required to attend long-term follow-up including audiograms and provide written informed consent.

COSTAR (CRUK/08/004; ISRCTN81772291) was approved by a National Research Ethics Committee (MREC 05/Q0801/183), sponsored by the Royal Marsden NHS Foundation Trust and undertaken in accordance with the principles of Good Clinical Practice.

The Institute of Cancer Research-Clinical Trials and Statistics Unit (ICR-CTSU) had overall responsibility for trial conduct, data collation, central statistical monitoring and statistical analyses. The trial was overseen by an independent Trial Steering Committee. An Independent Data Monitoring Committee (IDMC) reviewed emerging safety and efficacy data in confidence.

### 2.2 Randomisation

Patients were randomly assigned (1:1) to 3DCRT or CS-IMRT via a telephone call to the ICR-CTSU. Initially, treatment allocation used random permuted blocks with stratification by treatment centre and intended dose (60Gy or 65Gy). From 14 Apr 2011, after 48 patients were recruited, minimisation with a random element (balancing by centre and dose) was used. Clinicians were not masked to treatment allocation.

### 2.3 Procedures

**Fig. 1:**
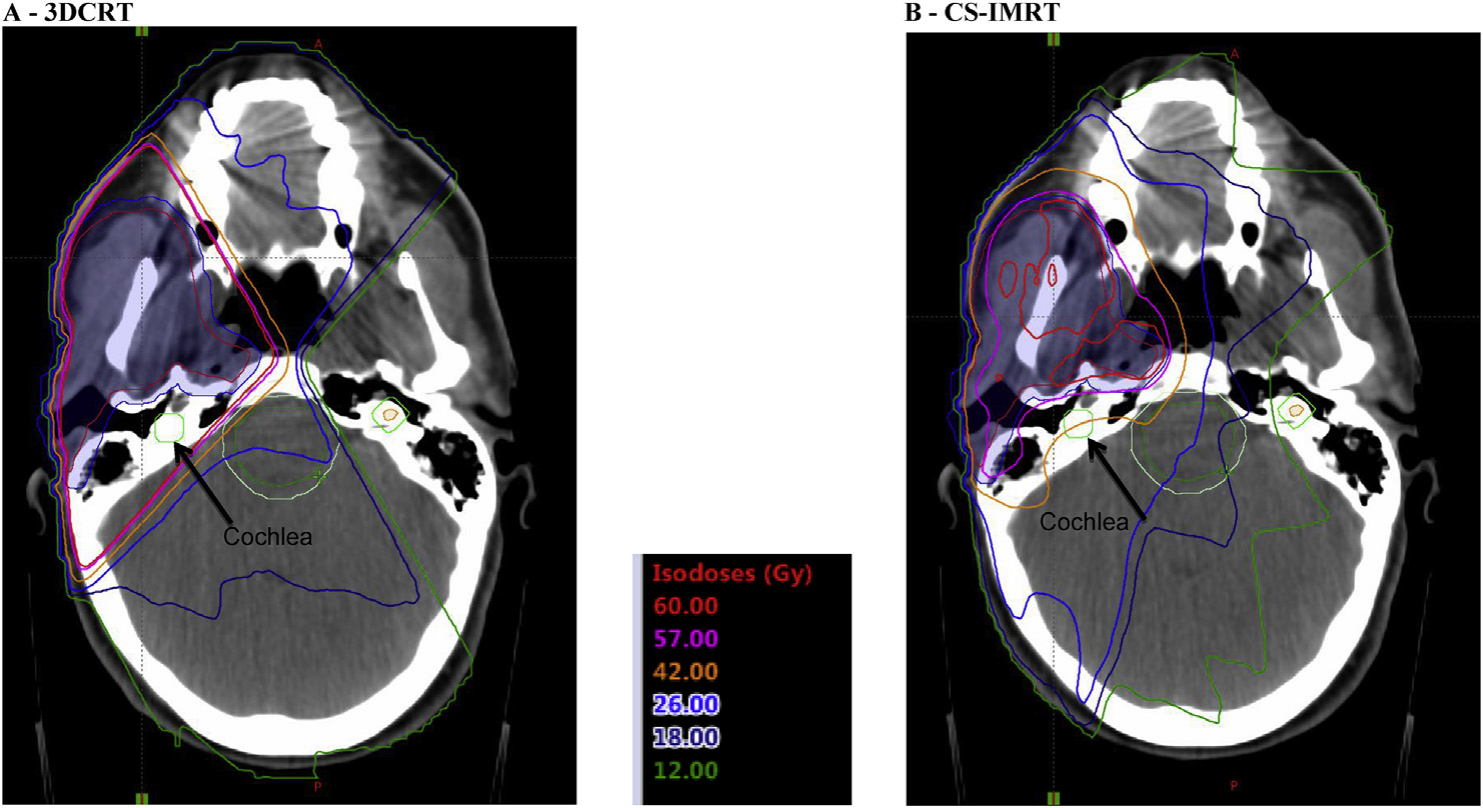
Typical dose distributions for 3DCRT (A) and CS-IMRT (B) demonstrating reduction of radiation dose to cochlea. 3DCRT, 3-dimensional conformal radiotherapy; CS-IMRT, cochlear-sparing intensity-modulated radiotherapy.

After obtaining fully informed written consent, all patients underwent radiotherapy treatment outlining and planning, according to the target volume definition guidelines detailed in Web Appendix 1. Trial quality assurance of the radiotherapy procedures was undertaken as part of Radiotherapy Trials Quality Assurance programme. Before trial entry, every centre completed a facility questionnaire and a process document. These defined each centre's equipment and methods for CT simulation, treatment planning, delivery and verification and patient set-up verification (Web appendix 2 p1-2). In addition, an outlining and planning benchmark case was submitted by each centre (Fig. 1). All documents and benchmarks were reviewed by the quality assurance physicist and Chief Investigator for the quality and compliance; resubmission based on detailed feedback was requested when necessary. Once a centre was approved for trial entry, the first three patients were submitted for a prospective case review by the Chief Investigator and physicist. Outlines and plans were modified when necessary before patients were treated. Dosimetry audit visits were conducted to each centre that entered patients to assess the accuracy of treatment delivery.

Baseline audiograms, a balance assessment and patient-reported quality of life (QoL) were measured before treatment. Acute toxicity was measured weekly during radiotherapy and up to 8 weeks afterwards using the National Cancer Institute Common Toxicity Criteria, version 3 (CTCAE, v3.0) [16]. Late radiation toxicity was measured at 3, 6, 12, 18, 24, 36, 48 and 60 months from the end of radiotherapy using the CTCAE, v3.0, and the Late Effects of Normal Tissues, Subjective Objective Management Analytic (LENT-SOMA) [17] scoring systems. Patient-reported outcomes (PROs) were measured using the European Organisation for Research and Treatment of Cancer QLQ-C30 instrument [18], the associated head and neck–specific module HN35 and the Glasgow Hearing Aid Benefit Profile Questionnaire (GHABP) [19]. Questionnaire booklets were completed in clinic before randomisation and at 6, 12, 24 and 60 months from the end of radiotherapy.

Bilateral pure-tone audiograms and a balance assessment were carried out before radiotherapy, at 6 and 12 months and 2 and 5 years after. Standard pure tone audiometry was used [20]. Audiograms were obtained for bone and air conduction thresholds and reported at 4000 Hz. Balance function was documented using the Romberg test, Unterberger's step test and the head thrust test [21], [22], [23].

Independent central review of audiograms was performed by Prof. Linda Luxon (Emeritus Professor of Audiovestibular Medicine and Consultant Neuro-otologist at the UCL Ear Institute) after the last patient randomised reached the 12-month time point, with change in conductive and/or sensorineural hearing loss between baseline and 12 months categorised as no change, mild (26–40 dB), moderate (41–60 dB) or severe (>61 dB) [24].

### 2.4 Outcomes

The primary end-point was a reduction in sensorineural hearing loss measured by masked bone conduction at 4000 Hz of ≥10 dB in the cochlea ipsilateral to the parotid tumour between baseline and 12 months; 12 months was selected *a priori* as a clinically appropriate time at which to make a valid assessment of late effects on hearing [12], [13], [14].

Secondary end-points were auditory assessment at 6 and 24 months, acute and late side-effects, patient-reported QoL and hearing outcomes, balance assessment, time to recurrence (TTR) and overall survival (OS).

### 2.5 Statistical analysis

Previous studies suggested the incidence of sensorineural hearing loss from 3DCRT to be 40–60% [9], [12]. Eighty-four patients were required to detect a reduction from 40% to 10% in the proportion suffering sensorineural hearing loss (90% power, two-sided 5% significance level).

On 14 March 2012, the IDMC recommended an increase in the sample size to 110 to maintain statistical power because of a lower than expected proportion of patients evaluable for the primary end-point.

Analysis was by intention-to-treat, including all patients with both baseline and 12-month masked bone conduction threshold assessment. The proportion of patients with reduction in bone conduction threshold of ≥10 dB was compared using a Fisher's exact test. Odds ratios (ORs) for hearing loss were calculated using logistic regression (OR<1 in favour of CS-IMRT). Unadjusted and adjusted analyses for sex, grade of differentiation, intended treatment dose, baseline ipsilateral bone conduction threshold and age at randomisation were performed. Hearing loss by bone and air conduction testing was summarised by treatment group using medians and interquartile ranges (IQRs) at each time point with centrally assessed categorical hearing loss compared using the Chi-squared test for trend.

Rates of any grade and grade ≥3 acute and late side-effects at 12 months were compared using Fisher's exact tests. To allow for multiple testing, a significance level of 1% was used for all secondary toxicity and QoL end-points.

TTR was calculated from randomisation to date of disease recurrence, or death from parotid cancer, censored at the second primary cancer diagnosis, death from other cause or date last seen. OS was calculated from randomisation to death from any cause, censored at the date last seen. Treatment groups were compared by log-rank test and hazard ratios (HRs) with 95% confidence intervals (CIs) obtained from Cox proportional hazards models with HR < 1 favouring CS-IMRT. The proportionality assumption of the Cox model held when tested with Schoenfeld residuals.

Analyses are based on a database snapshot taken on May 18, 2016, and were performed using STATA, v13.

## 3 Results

### 3.1 Patients

**Fig. 2:**
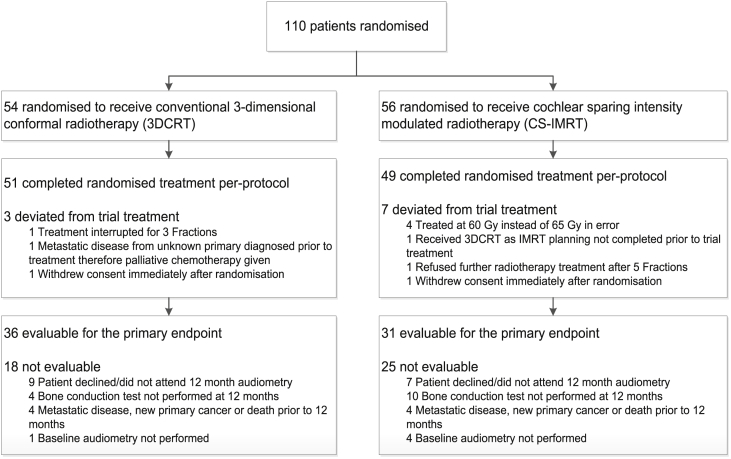
CONSORT diagram.

Between Aug 2008 and Feb 2013, 110 patients were randomised (3DCRT: 54; CS-IMRT: 56) (Fig. 2) from 22 UK radiotherapy centres (Web appendix 2 TableWA1). Median age at randomisation was 58 years (range 18–88) and 58/110 (53%) patients were male; 99/110 (90%) patients had R1/R2 resection status and received 65Gy/30f (Table 1). Randomised groups were well balanced for the tumour stage and grade. Median follow-up in living patients was 49.9 months (IQR 37.7–61.9).

**Table 1:** Baseline characteristics and treatment details.

	3DCRT (N = 54)	CS-IMRT (N = 56)
**Mean age at randomisation (years)**	59 (19–88)	57 (20–87)
**Sex**		
Male	31 (57%)	27 (48%)
Female	23 (43%)	29 (52%)
**Resection margin status**		
R0 (clear margins >5 mm)	6 (11%)	3 (5%)
R1 (margins 1–5 mm)	15 (28%)	14 (25%)
R2 (margins <1 mm)	32 (59%)	38 (68%)
Unknown	1 (2%)	1 (2%)
**Tumour grade**		
High	23 (43%)	18 (32%)
Intermediate	9 (17%)	11 (20%)
Low	16 (30%)	21 (38%)
Unknown	6 (11%)	6 (11%)
**T-stage**		
T1	16 (30%)	16 (29%)
T2	15 (28%)	22 (39%)
T3	7 (13%)	8 (14%)
T4	12 (22%)	9 (16%)
Unknown	4 (7%)	1 (2%)
**N-stage**		
N0	32 (59%)	37 (66%)
N1	4 (7%)	7 (13%)
N2	14 (26%)	9 (16%)
Unknown	4 (7%)	3 (5%)
**Radiotherapy dose (Gy)**		
Median dose to the primary tumour and involved nodes	65.0 (64.9–65.0; 51)	65.0 (65.0–65.0; 54)
R0 patients	59.1 (58.0 - 60.0; 6)	60.3 (60.0 - 64.9; 3)
R1/R2 patients	65.0 (65.0 - 65.0; 45)	65.0 (65.0 - 65.0; 51)
Median dose to elective nodes^a^	52.0 (50.0–60.0; 19)	54.4 (54.0–55.4; 20)
Mean dose to contralateral cochlea	6.1 (2.9–8.8; 51)	8.3 (6.6–9.3; 54)
Mean dose to ipsilateral cochlea^b^^c^	56.2 (44.6–61.0; 51)	35.7 (30.0–39.0; 54)
Mean dose to contralateral parotid	4.8 (3.0–9.8; 51)	10.8 (9.1–13.5; 54)
Maximum dose to brain stem	35.7 (33.3–40.9; 51)	42.9 (38.6–48.0; 54)
Maximum dose to spinal cord	37.3 (32.5–39.0; 51)	40.0 (37.3–42.2; 54)

3DCRT, 3-dimensional conformal radiotherapy; CS-IMRT, cochlear-sparing intensity-modulated radiotherapy; IQR, interquartile range.

Data are mean (range), n (%) or median (IQR; n).

a Only includes patients who received elective nodal irradiation.

b Mann–Whitney test p < 0·0001.

c If restricting to patients evaluable for the primary endpoint, mean doses to the ipsilateral cochlea are as follows: 3DCRT n = 36, median = 55.4, IQR 41.8–61.9; CS-IMRT n = 31, median = 36.1, IQR 31.9–39.2.

### 3.2 Radiotherapy

Radiotherapy doses are detailed in Table 1. PTV coverage was not adversely affected by cochlear sparing in the CS-IMRT arm (Table 1). Median dose to the ipsilateral cochlea was 56.2Gy with 3DCRT and 35.7Gy with CS-IMRT (Mann–Whitney p < 0.0001). An additional analysis of the accuracy of the cochlea outlining was performed on all patients after the trial was completed. Maximum doses to the spinal cord and brain stem and mean dose to the contralateral parotid gland were higher with CS-IMRT than with 3DCRT (p = 0.0022, p = 0.0001 and p < 0.0001, respectively) but were within tolerances.

### 3.3 Hearing impairment and balance assessment

67/110 (61%) patients had paired baseline and 12-month (masked) bone conduction measurements and were evaluable for the primary end-point (Table 2). At 12 months after radiotherapy, 25/67 (37%) patients had ≥10 dB sensorineural hearing loss; 3DCRT: 14/36 (39%), CS-IMRT: 11/31 (35%), p = 0.81 (Table 2). Unadjusted and adjusted ORs for hearing loss were 0.86 (95% CI 0.32–2.34) and 0.80 (95% CI 0.27–2.37), respectively. Proportion with hearing loss ≥20 dB was 19% with 3DCRT and 16% with CS-IMRT, and loss ≥30 dB was 8% with 3DCRT and 3% with CS-IMRT.

**Table 2:** Audiometry and vestibular function at 12 months after radiotherapy (primary end-point).

Audiometry	N with paired data	Threshold level (dB) at 4000 Hz Median (IQR)			N with ≥10 dB loss	%	p-value for 3DCRT versus CS-IMRT^b^
		Pre-radiotherapy	12 months after radiotherapy	Change^a^			
**Bone conduction–Ipsilateral ear**^c^							
3DCRT	36	30 (10–42.5)	40 (15–50)	5 (-5–15)	14	38.9	0.81
CS-IMRT	31	20 (10–45)	35 (15–55)	5 (0–10)	11	35.5	
**Air conduction–Ipsilateral ear**							
3DCRT	42	35 (15–55)	50 (20–70)	10 (0–20)	23	54.8	0.53
CS-IMRT	44	25 (10–45)	40 (15–62.5)	5 (0–22.5)	21	47.7	
**Bone conduction–Contralateral ear**							
3DCRT	32	22.5 (10–50)	27.5 (15–50)	5 (0–10)	9	28.1	0.76
CS-IMRT	23	15 (10–40)	20 (5–45)	0 (-5–5)	5	21.7	
**Air conduction–Contralateral ear**							
3DCRT	42	25 (15–55)	27.5 (20–55)	5 (0–5)	9	21.4	0.26
CS-IMRT	43	25 (10–45)	20 (10–50)	0 (-5–0)	5	11.6	

Balance assessment	N with test result at 12 months	N with abnormal result	%
**Romberg test**			
3DCRT	36	2	5.6
CS-IMRT	39	2	5.1
**Unterberger's test**			
3DCRT	36	4	11.1
CS-IMRT	37	7	18.9
**Head thrust test**			
3DCRT	33	2	6.1
CS-IMRT	34	2	5.9

3DCRT, 3-dimensional conformal radiotherapy; CS-IMRT, cochlear-sparing intensity-modulated radiotherapy; IQR, interquartile range.

Only includes baseline/12-month data on patients with data from both time points available.

a Calculated as hearing level at 12 months after radiotherapy (RT) minus threshold level before RT. A change greater than zero indicates a loss of hearing from pre-RT to 12 months post-RT.

b p-value from Fisher's exact test comparing proportions with ≥10 dB loss in 3DCRT and CS-IMRT groups.

c Primary end-point.

For air conduction thresholds at 12 months, a loss of ≥10 dB was seen in 44/86 (51%) patients (3DCRT: 23/42 [55%]; CS-IMRT: 21/44 [48%]; p = 0.53). No statistically significant differences in hearing were seen when measured by bone or air conduction thresholds at either 6 or 24 months (Web appendix 2 Tables WA2 and WA3) or by centrally assessed hearing loss (Web appendix 2 Table WA4). Balance was not affected by treatment with no statistically significant differences seen (Table 2).

### 3.4 Acute and late radiation toxicity

There were no statistically significant differences between 3DCRT and CS-IMRT for any acute side-effects during and up to 8 weeks after radiotherapy (Table 3).

**Table 3:** Acute and late toxicity.

	3DCRT											CS-IMRT											p-value for 3DCRT vs CS-IMRT	
	N	Grade 0		Grade 1		Grade 2		Grade 3		Grade 4		N	Grade 0		Grade 1		Grade 2		Grade 3		Grade 4			
		n	%	n	%	n	%	n	%	n	%		n	%	n	%	n	%	n	%	n	%	≥Grade 1	≥Grade 3
**Acute side-effects (CTCAE)**^a^																								
Hearing	43	19	44%	13	30%	9	21%	2	5%	0	0%	50	30	60%	9	18%	8	16%	3	6%	0	0%	0.15	>0.99
Otitis–external ear	51	11	22%	20	39%	18	35%	2	4%	0	0%	54	19	35%	21	39%	14	26%	0	0%	0	0%	0.14	0.23
Otitis–middle ear	46	19	41%	17	37%	9	20%	1	2%	0	0%	51	27	53%	16	31%	7	14%	1	2%	0	0%	0.31	>0.99
Tinnitus	51	18	35%	0	0%	29	57%	4	8%	0	0%	53	28	53%	0	0%	24	45%	1	2%	0	0%	0.08	0.20
Pain (otalgia)	51	14	27%	20	39%	15	29%	2	4%	0	0%	53	24	45%	17	32%	11	21%	1	2%	0	0%	0.07	0.61
Radiation dermatitis	51	1	2%	1	2%	34	67%	15	29%	0	0%	55	1	2%	9	16%	34	62%	11	20%	0	0%	>0.99	0.37
Alopecia	51	6	12%	19	37%	26	51%	0	0%	0	0%	55	2	4%	15	27%	31	56%	7	13%	0	0%	0.31	–
Pharyngeal dysphagia	51	3	6%	16	31%	27	53%	5	10%	0	0%	55	2	4%	15	27%	31	56%	7	13%	0	0%	0.67	0.76
Fatigue	51	1	2%	17	33%	24	47%	9	18%	0	0%	55	3	5%	20	36%	26	47%	6	11%	0	0%	0.62	0.41
Mucositis	51	0	0%	7	14%	30	59%	14	27%	0	0%	55	1	2%	6	11%	33	60%	15	27%	0	0%	>0.99	>0.99
Pain (other)	51	0	0%	11	22%	26	51%	14	27%	0	0%	55	2	4%	21	38%	19	35%	13	24%	0	0%	0.50	0.66
Mouth dryness	51	1	2%	16	31%	31	61%	3	6%	0	0%	55	2	4%	19	35%	32	58%	2	4%	0	0%	>0.99	0.67
Salivary gland changes	51	5	10%	18	35%	26	51%	2	4%	0	0%	55	3	5%	17	31%	34	62%	1	2%	0	0%	0.48	0.61
**Late side-effects (CTCAE)**^b^																								
Hearing	49	13	27%	15	31%	14	29%	6	12%	1	2%	54	14	26%	20	37%	11	20%	5	9%	4	7%	>0.99	0.79
Otitis–external ear	50	33	66%	13	26%	3	6%	1	2%	0	0%	54	34	63%	16	30%	4	7%	0	0%	0	0%	0.84	0.48
Otitis–middle ear	50	33	66%	14	28%	2	4%	1	2%	0	0%	54	36	67%	16	30%	2	4%	0	0%	0	0%	>0.99	0.48
Tinnitus	50	21	42%	0	0%	28	56%	1	2%	0	0%	54	28	52%	2	4%	20	37%	4	7%	0	0%	0.33	0.37
Pain (otalgia)	50	32	64%	12	24%	5	10%	1	2%	0	0%	54	38	70%	15	28%	1	2%	0	0%	0	0%	0.54	0.48
Skin pigmentation	49	22	45%	24	49%	3	6%	0	0%	0	0%	54	29	54%	23	43%	2	4%	0	0%	0	0%	0.43	–
Skin atrophy	50	25	50%	24	48%	1	2%	0	0%	0	0%	54	29	54%	24	44%	1	2%	0	0%	0	0%	0.84	–
Skin fibrosis	50	18	36%	27	54%	5	10%	0	0%	0	0%	54	22	41%	30	56%	2	4%	0	0%	0	0%	0.69	–
Mucous membranes–functional	50	35	70%	10	20%	4	8%	1	2%	0	0%	54	31	57%	18	33%	5	9%	0	0%	0	0%	0.22	0.48
Mucous membranes–clinical examination	50	36	72%	12	24%	2	4%	0	0%	0	0%	54	39	72%	13	24%	2	4%	0	0%	0	0%	>0.99	–
Mouth dryness	50	8	16%	29	58%	11	22%	2	4%	0	0%	54	3	6%	39	72%	11	20%	1	2%	0	0%	0.11	0.61
Salivary gland changes	50	13	26%	27	54%	10	20%	0	0%	0	0%	54	12	22%	37	69%	4	7%	1	2%	0	0%	0.82	>0.99
Osteonecrosis	50	49	98%	1	2%	0	0%	0	0%	0	0%	54	53	98%	1	2%	0	0%	0	0%	0	0%	>0.99	–
Trismus	50	32	64%	15	30%	3	6%	0	0%	0	0%	54	28	52%	23	43%	3	6%	0	0%	0	0%	0.24	–
Fatigue	50	29	58%	13	26%	5	10%	3	6%	0	0%	53	28	53%	21	40%	4	8%	0	0%	0	0%	0.69	0.11
**Late side-effects (LENT-SOMA)**^c^																								
Ear	50	3	6%	15	30%	10	20%	16	32%	6	12%	54	6	11%	20	37%	10	19%	10	19%	8	15%	0.49	0.32
*Subjective hearing*	*50*	*8*	*16%*	*16*	*32%*	*18*	*36%*	*7*	*14%*	*1*	*2%*	*54*	*11*	*20%*	*23*	*43%*	*13*	*24%*	*6*	*11%*	*1*	*2%*	*0.62*	*0.78*
Mucosa (oral and pharyngeal)	50	5	10%	22	44%	12	24%	11	22%	0	0%	54	9	17%	27	50%	11	20%	5	9%	2	4%	0.40	0.30
Salivary gland	50	2	4%	20	40%	17	34%	10	20%	1	2%	54	3	6%	21	39%	23	43%	7	13%	0	0%	>0.99	0.30
*Subjective xerostomia*	*50*	*5*	*10%*	*23*	*46%*	*18*	*36%*	*3*	*6%*	*1*	*2%*	*54*	*3*	*6%*	*30*	*56%*	*18*	*33%*	*3*	*6%*	*0*	*0%*	*0.48*	*0.71*
Mandible	50	23	46%	6	12%	17	34%	4	8%	0	0%	54	22	41%	14	26%	18	33%	0	0%	0	0%	0.69	0.05
Teeth	49	39	80%	8	16%	2	4%	0	0%	0	0%	54	41	76%	8	15%	3	6%	2	4%	0	0%	0.81	0.50
Spinal cord	50	45	90%	3	6%	1	2%	1	2%	0	0%	54	47	87%	5	9%	2	4%	0	0%	0	0%	0.76	0.48
Skin	50	3	6%	24	48%	17	34%	6	12%	0	0%	54	3	6%	29	54%	12	22%	10	19%	0	0%	>0.99	0.42

3DCRT, 3-dimensional conformal radiotherapy; CS-IMRT, cochlear-sparing intensity-modulated radiotherapy.

a Maximum Common Toxicity Criteria (v3.0) score during and up to 8 weeks after radiotherapy, Table 3.

b Maximum Common Toxicity Criteria (v3.0) score between 3 and 60 months after radiotherapy.

c Maximum LENT-SOMA (Late Effects of Normal Tissues, Subjective Objective Management Analytic) score between 3 and 60 months after radiotherapy. For LENT-SOMA scales, the maximum of the subjective, objective, management and analytic component scores was used.

Clinician-assessed late radiation toxicity confirmed the primary end-point findings with no difference in the proportion of patients with any CTCAE-grade hearing toxicity at 12 months (3DCRT: 17/39 [44%]; CS-IMRT: 22/47 [47%]). Dry mouth (any grade) at 12 months was more prevalent in patients receiving CS-IMRT (38/48, 79%) than 3DCRT (22/43, 51%), p = 0.008. Similar (but not statistically significant) differences in dry mouth were also seen at 18 and 24 months. There were no statistically significant differences in other late radiation toxicity scores according to the CTCAE (Table 3), either at 12 months or when considering the maximum grade reported during follow-up. No statistically significant differences in LENT-SOMA hearing domains were seen at 12 months. However, there was a numerically higher incidence of salivary gland toxicity with CS-IMRT (37/48, 77%) than with 3DCRT (27/43, 63%), p = 0.17.

### 3.5 PROs and QoL

**Fig. 3:**
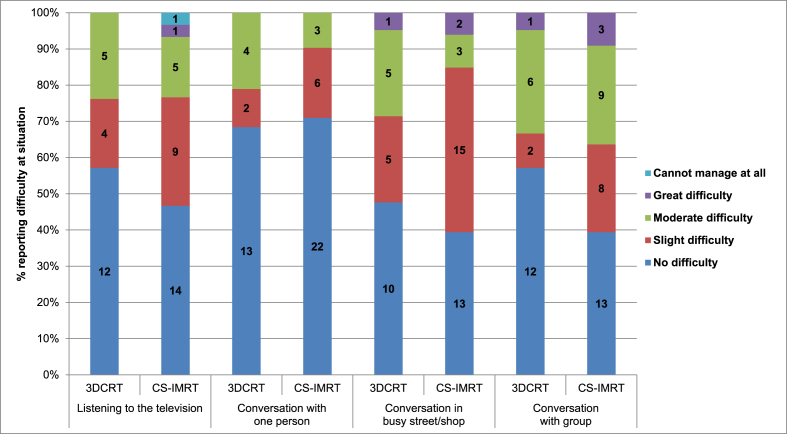
Glasgow Hearing Aid Benefit Profile at 12 months after radiotherapy by randomised treatment group.

No statistically significant differences between treatment groups were seen in any of the four domains of the hearing-specific GHABP (Fig. 3) or in general cancer-related QoL using QLQ-C30 or QLQ-HN35 (Web appendix 2 Table WA5).

### 3.6 Disease-related outcomes

21/110 (19.1%) patients had a TTR event (3DCRT: 9/54 [16.7%], CS-IMRT: 12/56 [21.4%]). Of these, four had a locoregional recurrence as the first event (3DCRT: 2; CS-IMRT: 2), 13 had a distant recurrence (3DCRT: 6, CS-IMRT: 7) and four died from parotid cancer or unknown cause without prior recurrence reported (3DCRT: 1, CS-IMRT: 3). Two-year TTR event-free rate was 88% (95% CI 75%–94%) with 3DCRT and 88% (95% CI 76%–95%) with CS-IMRT (p = 0.75), HR 1.16 (95% CI 0.48–2.79) (Web appendix 2 Figure WA1A).

21/110 (19.1%) patients have died (3DCRT: 11/54 [20.4%], CS-IMRT: 10/56 [17.9%]). Of these, 12 died from parotid cancer (3DCRT: 7, CS-IMRT: 5). Two-year OS was 82% (95% CI 69%–90%) with 3DCRT and 92% (95% CI 81%–97%) with CS-IMRT (p = 0.72), HR 0.85 (95% CI 0.36–2.01), Web appendix 2 Figure WA1B.

## 4 Discussion

The radiation tolerance of the cochlea is thought to be a mean dose of 40–45Gy [25], [26]. The CS-IMRT technique evaluated in COSTAR achieved a mean cochlea dose of 35.7Gy, and 75% of patients randomised to CS-IMRT received a mean dose of <39Gy (Table 1). Despite reducing the cochlea dose to less than the tolerance dose, no statistically significant difference in masked bone conduction threshold at 4000 Hz was demonstrated between CS-IMRT and 3DCRT. Secondary end-points for which a greater proportion of patients were evaluable supported the primary end-point results.

An unexpected finding was that the incidence of late xerostomia appeared to be higher in patients receiving CS-IMRT. This could be due to a low-dose bath of radiation in the oral cavity and oropharynx adversely affecting the function of minor salivary glands in the palatal mucosa and causing dry mouth. The contralateral parotid dose was also higher with CS-IMRT compared with 3DCRT (Table 1).

COSTAR is the only randomised controlled trial to investigate CS-IMRT. The incidence of hearing loss seen with 3DCRT was consistent with the 30–50% [3], [4], [27] reported in studies not using IMRT. Theunissen *et al.* (n = 36) and Zuur *et al.* (n = 101) attempted to minimise cochlear dose (mean 17.8Gy, median 11.4Gy, respectively) using IMRT and measured hearing before and after treatment [28], [29]. Mean hearing deterioration in both studies was small and non-significant for frequencies 1000–4000 Hz. However, the incidence of hearing loss of >10 dB was 36% and 13%, respectively.

A key limitation of COSTAR was that 40% of patients were not evaluable for the primary end-point because of audiometry not being performed or masked bone conduction thresholds not being obtained. The observed 67 evaluable patients provide reduced but acceptable power of 80%. The proportion of patients with ≥10 dB loss in each treatment group was similar suggesting it is unlikely that a clinically relevant difference was missed because of the lack of statistical power.

A more likely reason for the hearing loss seen despite CS-IMRT is that the previously accepted cochlear tolerance of 40–45Gy is too high. If this is the case, then it may be necessary to reduce the cochlea dose much further to maintain cochlea function. Owing to the short distance from the edge of the parotid PTV to the cochlea, this is unlikely to be possible using IMRT and it may be that cochlear sparing to very low doses is better achieved using more conformal techniques such as proton therapy [30].

It is possible, although unlikely, that the mean cochlea dose reported in the study is not a true reflection of the cochlea dose received. A number of factors could have contributed to this. First, the cochlea could have been incorrectly localised by the treating physician. On review of the quality assurance CT data sets, this was excluded. Second, patient movement could have resulted, by interfraction motion, in a different dose being delivered to the cochlea than was estimated during treatment planning. This also seems an unlikely explanation, given that a planning risk volume margin of 3 mm was added to the cochlea organ at risk and that set-up was checked weekly throughout treatment.

Use of chemotherapy in COSTAR was not permitted. In locally advanced mucosal squamous cell carcinomas of the head and neck, where concomitant platinum-based chemotherapy is the standard of care, auditory toxicity is even more common. Cochlear sparing could be tested in a randomised trial in this group, although it would be difficult to control for chemotherapy intensity.

## 5 Conclusions

CS-IMRT reduced the radiation dose below the accepted tolerance of the cochlea. CS-IMRT did not result in statistically or clinically significant reductions in the proportion of patients with measured or self-reported hearing loss in the ipsilateral ear at 12 months after radiotherapy and may increase patient-reported xerostomia.

## Authorship contributions

Christopher M Nutting, Sheerang Bhide, James P Morden and Emma Hall contributed in conception and design. Matthew Beasley, Shreerang Bhide, Emma De Winton, Mererid Evans, Lydia Fresco, Simon Gollins, Kevin Harrington, Catherine Lemon, Ruheena Mendes, Kate Newbold, Robin Prestwich, Martin Robinson, Paul Sanghera, Joanna Simpson and Muthiah Sivaramalingam provided study materials or patients. Christopher M Nutting, Matthew Beasley, Shreerang Bhide, Audrey Cook, Emma De Winton, Marie Emson, Mererid Evans, Lydia Fresco, Simon Gollins, Dorothy Gujral, Kevin Harrington, Mano Joseph, Catherine Lemon, Qurrat van den Blink, Ruheena Mendes, Aisha Miah, Kate Newbold, Robin Prestwich, Martin Robinson, Paul Sanghera, Joanna Simpson, Muthiah Sivaramalingam, Narayanan Nair Srihari, Mark Sydenham, Emma Wells and Stephanie Witts helped in the collection and assembly of data. Christopher M Nutting, James P Morden, Linda Luxon and Emma Hall involved in data analysis and interpretation. All authors helped in manuscript writing, approved the final version of the manuscript and are accountable for all aspects of the work.

## Conflict of interest statement

The authors declare no conflicts of interest.

## Funding

COSTAR was supported by Cancer Research UK (grant numbers C8262/A8963, C1491/A9895), trial reference number CRUK/08/004).
