# Supplementary material for: Results of a multicentre randomised controlled trial of cochlear-sparing intensity-modulated radiotherapy versus conventional radiotherapy in patients with parotid cancer (COSTAR; CRUK/08/004)
Source: Eur J Cancer. 2018 Nov;103:249–58. doi: 10.1016/j.ejca.2018.08.006 (PMC6202674; doi:10.1016/j.ejca.2018.08.006)
Supplement: Multimedia component 1 [file mmc1.docx]

**Web appendix 2**

**Radiotherapy procedures**

A thermoplastic immobilisation shell was constructed with the patient’s neck extended and contrast-enhanced CT scanning was performed using 2.5mm slices through the head and neck region in both treatment arms.

Clinical Target Volumes (CTVs) were delineated on each CT slice. CTV1 included the post-operative parotid bed and level II lymph nodes. This was defined as a volume extending from the zygomatic arch superiorly to the lower border of the hyoid bone inferiorly, the anterior border of the masseter muscle anteriorly and the surface of the temporal bone at the skull base posteriorly, the subcutaneous tissue laterally and to include the residual deep lobe of the parotid medially.

For patients requiring elective or post-operative nodal irradiation, CTV2 included ipsilateral levels III-V. These were contoured using the EORTC, GORTEC and RTOG endorsed consensus guidelines for the delineation of the CTV in the neck for patients with head and neck squamous cell carcinoma[^1^](#_ENREF_20).

Both cochleas, spinal cord, brainstem, contralateral parotid and lenses of both eyes were outlined as organs-at-risk (OARs). The mean dose constraints were: Ipsilateral cochlea – <40Gy for IMRT patients; Contralateral cochlea - <10Gy; Contralateral parotid - <20Gy; Spinal cord – Max dose 48Gy for all patients; Brian stem – Maximum dose 55Gy for all patients. A margin of 3-5mm was added to the CTVs and OARs to produce PTVs and planning organ-at-risk volumes (PRVs), respectively.

For patients treated with 3DCRT, anterior- and posterior-oblique isocentric 6MV photon fields were used to cover the PTV1. The other beam parameters were chosen to ensure an adequate coverage of the PTV and to keep the doses to the OARs within tolerance. Due to the shape of the PTV and the proximity of the cochlea to the posterior edge of PTV1, the cochlea was usually within the 90% isodose line (Fig 1A). In patients requiring neck irradiation, this was matched inferiorly to the superior fields using a single isocentre technique. A radiation dose of 60Gy in 30 fractions once daily over 6 weeks was prescribed in patients with an R0 resection margin. 65Gy in 30 fractions was prescribed if there were close margins (R1 resection) or residual macroscopic disease (R2 resection). A dose of 50Gy in 25 fractions was prescribed to elective nodal sites and 60Gy in 30 fractions for post-operative adjuvant radiotherapy.

CS-IMRT was inverse-planned in each treating centre using the local radiotherapy planning system. Multiple beams were used to obtain uniform coverage of the PTV and satisfy the dose constraints to the OARs (Fig 1B). Arc therapy was not available in the UK during trial recruitment. For IMRT plan optimisation, the cochlea PRV was assigned a constraint to reduce the radiation dose to <40Gy. Treatment was prescribed to the median dose. The prescription dose to PTVs was the same as for the 3DCRT plans (60Gy or 65Gy in 30 fractions as above).

Patient position was monitored during treatment by electronic portal imaging or cone-beam CT every day for the first week and weekly thereafter. Set-up tolerance of up to 3-5mm was accepted, and corrections were made for any systematic set-up errors.

**References**

1. Gregoire V, Levendag P, Ang KK, et al: CT-based delineation of lymph node levels and related CTVs in the node-negative neck: DAHANCA, EORTC, GORTEC, NCIC,RTOG consensus guidelines. Radiother Oncol 69:227-36, 2003

**Web appendix 2 Table WA1 – List of COSTAR recruiting clinicians**

| **Recruiting site**  **(patients recruited)** | **Recruiting clinicians**  **(patients recruited)** |
| --- | --- |
| Royal Marsden Hospital, Sutton (15) | Christopher Nutting (6)  Shreerang Bhide (5)  Kate Newbold (4) |
| Royal Marsden Hospital, London (14) | Christopher Nutting (12)  Kevin Harrington (2) |
| Mount Vernon Hospital (9) | Catherine Lemon (7)  Kate Goodchild (2) |
| Weston Park Hospital, Sheffield (8) | Om Prakash Purohit (4)  Martin Robinson (3)  Bernadette Foran (1) |
| Glan Clwyd Hospital, Rhyl (7) | Rekha Neupane (3)  Win Soe (2)  Simon Gollins (2) |
| Royal Preston Hospital (7) | Muthiah Sivaramalingham (7) |
| University Hospital Coventry, Coventry (7) | Lydia Fresco (5)  Andrew Chan (2) |
| Queen Elizabeth Hospital, Birmingham (6) | Andrew Hartley (4)  John Glaholm (1)  Paul Sanhera (1) |
| St James’s University Hospital, Leeds (6) | Robin Prestwich (3)  Catherine Coyle (3) |
| Velindre Cancer Centre (6) | Mererid Evans (3)  Nachiappan Palaniappan (2)  Laura Moss (1) |
| Bristol Haematology and Oncology Centre (4) | Matthew Beasley (4) |
| Royal Sussex County Hospital (4) | Richard Simcock (3)  Joanna Simpson (1) |
| New Cross Hospital, Wolverhampton (3) | Caroline Brammer (3) |
| Northampton General Hospital (3) | Gerard Andrade (3) |
| Royal United Hospital, Bath (3) | Emma De Winton (3) |
| Norfolk & Norwich Hospital (2) | Tom Roques (1)  Craig Martin (1) |
| Castle Hill Hospital, Cottingham (1) | Abdel Hamid (1) |
| Cheltenham General Hospital (1) | Stephen Shepherd (1) |
| Leicester Royal Infirmary (1) | David Peel (1) |
| Royal Shrewsbury Hospital (1) | Ravi Prashant (1) |
| Southampton General Hospital (1) | Chris Baughan (1) |
| University College Hospital, London (1) | Ruheena Mendes (1) |

**Web appendix 2 Table WA2 - Audiometry at 6 months after radiotherapy**

|  | **N with**  **paired data** | **Threshold level (dB) at 4000Hz Median (IQR)** | | | **N with ≥10 dB loss** | **%** | **p-value for 3DCRT vs. CS-IMRT^2^** |
| --- | --- | --- | --- | --- | --- | --- | --- |
|  |  | **Pre-RT** | **6 months post-radiotherapy** | **Change^1^** |  |  |  |
| **Bone conduction - Ipsilateral ear** |  |  |  |  |  |  |  |
| 3DCRT | 36 | 30 (10 - 40) | 30 (15 – 47.5) | 0 (0 - 10) | 11 | 30.6 | >0.99 |
| CS-IMRT | 38 | 20 (10 - 45) | 22.5 (15 - 45) | 0 (0 - 10) | 12 | 31.6 |  |
|  |  |  |  |  |  |  |  |
| **Air conduction - Ipsilateral ear** |  |  |  |  |  |  |  |
| 3DCRT | 40 | 35 (15 - 50) | 45 (20 - 75) | 5 (0 -15) | 16 | 40.0 | 0.51 |
| CS-IMRT | 45 | 25 (10 - 45) | 35 (20 - 60) | 5 (0 - 20) | 22 | 48.9 |  |
|  |  |  |  |  |  |  |  |
| **Bone conduction - Contralateral ear** |  |  |  |  |  |  |  |
| 3DCRT | 33 | 10 (5 - 35) | 22 (10 - 30) | 0 (0 - 5) | 6 | 18.2 | 0.76 |
| CS-IMRT | 31 | 15 (10 - 40) | 25 (10 - 40) | 0 (-5 - 5) | 7 | 22.6 |  |
|  |  |  |  |  |  |  |  |
| **Air conduction - Contralateral ear** |  |  |  |  |  |  |  |
| 3DCRT | 40 | 27.5 (10 - 55) | 30 (10 - 50) | 0 (0 - 5) | 8 | 20.0 | 0.22 |
| CS-IMRT | 44 | 25 (10 - 45) | 22.5 (15 - 45) | 0 (-7.5 - 5) | 4 | 9.1 |  |
|  |  |  |  |  |  |  |  |

Note: Only includes baseline/6 month data on patients with data from both time points available

^1^ Calculated as hearing level at 6 months post-RT minus threshold level pre-RT. A change greater than zero indicates a loss of hearing from pre-RT to 6 months post-RT.

^2^ p-value from Fisher’s exact test comparing proportions with ≥10 dB loss in 3DCRT and CS-IMRT groups

**Web appendix 2 Table WA3 - Audiometry at 24 months after radiotherapy**

|  | **N with**  **paired data** | **Threshold level (dB) at 4000Hz Median (IQR)** | | | **N with ≥10 dB loss** | **%** | **p-value for 3DCRT vs. CS-IMRT^2^** |
| --- | --- | --- | --- | --- | --- | --- | --- |
|  |  | **Pre-RT** | **24 months post-radiotherapy** | **Change^1^** |  |  |  |
| **Bone conduction - Ipsilateral ear** |  |  |  |  |  |  |  |
| 3DCRT | 26 | 30 (15 - 40) | 35 (15 - 50) | 5 (-5 - 15) | 11 | 42.3 | 0.38 |
| CS-IMRT | 25 | 20 (15 - 45) | 25 (10 - 45) | 0 (-5 - 10) | 7 | 28.0 |  |
|  |  |  |  |  |  |  |  |
| **Air conduction - Ipsilateral ear** |  |  |  |  |  |  |  |
| 3DCRT | 32 | 35 (15 - 45) | 45 (20 – 67.5) | 10 (0 – 22.5) | 19 | 59.4 | >0.99 |
| CS-IMRT | 36 | 30 (10 - 45) | 40 (20 - 70) | 10 (2.5 - 21) | 21 | 58.3 |  |
|  |  |  |  |  |  |  |  |
| **Bone conduction - Contralateral ear** |  |  |  |  |  |  |  |
| 3DCRT | 26 | 20 (10 - 40) | 27.5 (10 - 40) | 0 (-5 - 5) | 6 | 23.1 | 0.74 |
| CS-IMRT | 22 | 15 (10 - 45) | 22.5 (10 - 40) | -5 (-5 - 5) | 4 | 18.2 |  |
|  |  |  |  |  |  |  |  |
| **Air conduction - Contralateral ear** |  |  |  |  |  |  |  |
| 3DCRT | 34 | 30 (17 - 50) | 31 (20 - 45) | 0 (0 - 5) | 8 | 23.5 | 0.56 |
| CS-IMRT | 35 | 25 (10 - 45) | 30 (10 - 50) | 0 (-5 - 5) | 6 | 17.1 |  |
|  |  |  |  |  |  |  |  |

Note: Only includes baseline/24 month data on patients with data from both time points available

^1^ Calculated as hearing level at 24 months post-RT minus threshold level pre-RT. A change greater than zero indicates a loss of hearing from pre-RT to 24 months post-RT.

^2^ p-value from Fisher’s exact test comparing proportions with ≥10 dB loss in 3DCRT and CS-IMRT groups

**Web appendix 2 Table WA4 – Independent audiometry review**

A review of data was undertaken by Prof Linda Luxon (Emeritus Professor of Audiovestibular Medicine and Consultant Neuro-otologist at the UCL Ear Institute). Pair of audiograms (pre-treatment and 12 months) were assessed. Information on patient age and gender was provided to Prof Luxon, but not randomised treatment allocation. Hearing was scored separately for left and right ears, without knowledge of which was the treated side. Hearing loss at pre-treatment/12 months was scored as Normal/Mild/Moderate/Severe. Change between pre-treatment and 12 months was scored as No change/Mild loss/Moderate loss/Severe loss. Separate scores were given for conductive and sensori-neural loss.

The table below shows scores given by randomised treatment for the ipsilateral and contralateral ear.

|  | **3DCRT** | | **CS-IMRT** | | **Trend test  p-value*** |
| --- | --- | --- | --- | --- | --- |
|  | **N** | **%** | **N** | **%** |  |
| **Ipsilateral - Conductive** |  |  |  |  |  |
| **Pre-treatment** | **52** | **100.0** | **53** | **100.0** | 0.27 |
| Normal | 47 | 90.4 | 44 | 83.0 |  |
| Mild | 5 | 9.6 | 9 | 17.0 |  |
| Moderate | 0 | 0.0 | 0 | 0.0 |  |
| Severe | 0 | 0.0 | 0 | 0.0 |  |
|  |  |  |  |  |  |
| **12 months** | **36** | **100.0** | **40** | **100.0** | 0.50 |
| Normal | 23 | 63.9 | 29 | 72.5 |  |
| Mild | 11 | 30.6 | 9 | 22.5 |  |
| Moderate | 2 | 5.6 | 2 | 5.0 |  |
| Severe | 0 | 0.0 | 0 | 0.0 |  |
|  |  |  |  |  |  |
| **Change between pre-treatment and 12 months** | **39** | **100.0** | **40** | **100.0** | 0.81 |
| No change | 27 | 69.2 | 30 | 75.0 |  |
| Mild loss | 9 | 23.1 | 6 | 15.0 |  |
| Moderate loss | 3 | 7.7 | 4 | 10.0 |  |
| Severe loss | 0 | 0.0 | 0 | 0.0 |  |
|  |  |  |  |  |  |
| **Ipsilateral - Sensori-neural** |  |  |  |  |  |
| **Pre-treatment** | **52** | **100.0** | **54** | **100.0** | 0.28 |
| Normal | 19 | 36.5 | 26 | 48.2 |  |
| Mild | 13 | 25.0 | 11 | 20.4 |  |
| Moderate | 20 | 38.5 | 16 | 29.6 |  |
| Severe | 0 | 0.0 | 1 | 1.9 |  |
|  |  |  |  |  |  |
| **12 months** | **39** | **100.0** | **43** | **100.0** | 0.43 |
| Normal | 9 | 23.1 | 12 | 27.9 |  |
| Mild | 7 | 18.0 | 10 | 23.3 |  |
| Moderate | 21 | 53.9 | 20 | 46.5 |  |
| Severe | 2 | 5.1 | 1 | 2.3 |  |
|  |  |  |  |  |  |
| **Change between pre-treatment and 12 months** | **38** | **100.0** | **43** | **100.0** | 0.69 |
| No change | 23 | 60.5 | 27 | 62.8 |  |
| Mild loss | 10 | 26.3 | 12 | 27.9 |  |
| Moderate loss | 5 | 13.2 | 4 | 9.3 |  |
| Severe loss | 0 | 0.0 | 0 | 0.0 |  |
|  |  |  |  |  |  |
| **Contralateral - Conductive** |  |  |  |  |  |
| **Pre-treatment** | **52** | **100.0** | **51** | **100.0** | 0.98 |
| Normal | 49 | 94.2 | 47 | 92.2 |  |
| Mild | 2 | 3.9 | 4 | 7.8 |  |
| Moderate | 1 | 1.9 | 0 | 0.0 |  |
| Severe | 0 | 0.0 | 0 | 0.0 |  |
|  |  |  |  |  |  |
| **12 months** | **39** | **100.0** | **42** | **100.0** | 0.35 |
| Normal | 37 | 94.9 | 38 | 90.5 |  |
| Mild | 2 | 5.1 | 3 | 7.1 |  |
| Moderate | 0 | 0.0 | 1 | 2.4 |  |
| Severe | 0 | 0.0 | 0 | 0.0 |  |
|  |  |  |  |  |  |
| **Change between pre-treatment and 12 months** | **39** | **100.0** | **43** | **100.0** | 0.20 |
| No change | 39 | 100.0 | 41 | 95.4 |  |
| Mild loss | 0 | 0.0 | 1 | 2.3 |  |
| Moderate loss | 0 | 0.0 | 1 | 2.3 |  |
| Severe loss | 0 | 0.0 | 0 | 0.0 |  |
|  |  |  |  |  |  |
| **Contralateral - Sensori-neural** |  |  |  |  |  |
| **Pre-treatment** | **52** | **100.0** | **55** | **100.0** | 0.21 |
| Normal | 22 | 42.3 | 30 | 54.6 |  |
| Mild | 11 | 21.2 | 10 | 18.2 |  |
| Moderate | 19 | 36.5 | 14 | 25.5 |  |
| Severe | 0 | 0.0 | 1 | 1.8 |  |
|  |  |  |  |  |  |
| **12 months** | **40** | **100.0** | **44** | **100.0** | 0.42 |
| Normal | 15 | 37.5 | 22 | 50.0 |  |
| Mild | 10 | 25.0 | 7 | 15.9 |  |
| Moderate | 15 | 37.5 | 14 | 31.8 |  |
| Severe | 0 | 0.0 | 1 | 2.3 |  |
|  |  |  |  |  |  |
| **Change between pre-treatment and 12 months** | **40** | **100.0** | **44** | **100.0** | 0.28 |
| No change | 38 | 95.0 | 40 | 90.9 |  |
| Mild loss | 2 | 5.0 | 2 | 4.6 |  |
| Moderate loss | 0 | 0.0 | 2 | 4.6 |  |
| Severe loss | 0 | 0.0 | 0 | 0.0 |  |

*- Moderate and severe categories combined

**Web appendix 2 Table WA5 –** **Cross-sectional comparison of QoL at 12 months, by treatment group**

| **Subscale** | **3DCRT** | | | | **CS-IMRT** | | | | **T-test p-value** | **Mann-Whitney p-value** |
| --- | --- | --- | --- | --- | --- | --- | --- | --- | --- | --- |
|  | **n** | **Mean** | **99% CI** | | **n** | **Mean** | **99% CI** | |  |  |
|  |  |  | **Lower** | **Upper** |  |  | **Lower** | **Upper** |  |  |
| **QLQ-C30** |  |  |  |  |  |  |  |  |  |  |
| Global health status | 27 | 74.4 | 63.9 | 84.9 | 35 | 76.0 | 67.9 | 84.0 | 0.74 | 0.87 |
| Physical functioning | 27 | 87.7 | 77.5 | 97.9 | 35 | 88.0 | 80.8 | 95.1 | 0.96 | 0.42 |
| Role functioning | 27 | 86.4 | 74.6 | 98.3 | 35 | 89.0 | 79.7 | 98.4 | 0.63 | 0.54 |
| Emotional functioning | 27 | 82.1 | 71.2 | 93.0 | 35 | 80.7 | 72.3 | 89.1 | 0.78 | 0.61 |
| Cognitive functioning | 27 | 83.3 | 72.3 | 94.4 | 35 | 82.9 | 75.1 | 90.7 | 0.92 | 0.53 |
| Social functioning | 27 | 79.0 | 64.1 | 94.0 | 35 | 88.6 | 79.9 | 97.3 | 0.11 | 0.19 |
| Fatigue | 27 | 21.8 | 9.9 | 33.7 | 35 | 22.5 | 13.7 | 31.4 | 0.89 | 0.74 |
| Nausea and vomiting | 27 | 3.1 | -2.5 | 8.6 | 35 | 5.7 | -1.7 | 13.2 | 0.46 | 0.49 |
| Pain | 27 | 19.1 | 5.2 | 33.1 | 35 | 21.4 | 10.2 | 32.7 | 0.72 | 0.48 |
| Dyspnoea | 27 | 11.1 | -2.9 | 25.1 | 35 | 12.4 | 2.5 | 22.3 | 0.83 | 0.47 |
| Insomnia | 27 | 19.8 | 4.7 | 34.8 | 35 | 23.8 | 10.6 | 37.0 | 0.58 | 0.49 |
| Appetite loss | 27 | 16.0 | 3.6 | 28.5 | 35 | 12.4 | 1.1 | 23.6 | 0.55 | 0.30 |
| Constipation | 27 | 8.6 | -0.7 | 18.0 | 35 | 12.4 | 3.2 | 21.6 | 0.44 | 0.42 |
| Diarrhoea | 27 | 4.9 | -5.8 | 15.7 | 35 | 1.0 | -1.6 | 3.6 | 0.27 | 0.40 |
| Financial difficulties | 27 | 13.6 | -1.5 | 28.6 | 35 | 11.4 | 0.3 | 22.6 | 0.75 | 0.95 |
|  |  |  |  |  |  |  |  |  |  |  |
| **QLQ-HN35** |  |  |  |  |  |  |  |  |  |  |
| Pain | 27 | 17.0 | 8.2 | 25.8 | 35 | 16.0 | 8.7 | 23.2 | 0.80 | 0.89 |
| Swallowing | 27 | 6.5 | 1.3 | 11.6 | 35 | 6.0 | 1.0 | 10.9 | 0.84 | 0.71 |
| Senses | 27 | 20.4 | 5.1 | 35.7 | 35 | 19.0 | 9.2 | 28.9 | 0.84 | 0.78 |
| Speech | 27 | 9.9 | 0.1 | 19.7 | 35 | 9.2 | 2.5 | 16.0 | 0.87 | 0.72 |
| Social eating | 27 | 10.5 | 0.0 | 21.0 | 35 | 8.6 | 2.9 | 14.3 | 0.64 | 0.62 |
| Social contact | 27 | 9.4 | 2.0 | 16.8 | 35 | 4.4 | 0.8 | 8.0 | 0.08 | 0.21 |
| Sexuality | 26 | 25.6 | 8.1 | 43.2 | 29 | 19.0 | 2.4 | 35.6 | 0.45 | 0.52 |
| Teeth | 27 | 16.0 | 2.6 | 29.5 | 35 | 16.2 | 3.1 | 29.3 | 0.98 | 0.77 |
| Opening mouth | 27 | 25.9 | 11.7 | 40.2 | 35 | 29.5 | 16.2 | 42.8 | 0.62 | 0.65 |
| Dry mouth | 27 | 46.9 | 30.3 | 63.5 | 35 | 47.6 | 36.3 | 59.0 | 0.92 | 0.98 |
| Sticky saliva | 27 | 27.2 | 10.7 | 43.6 | 35 | 30.5 | 16.8 | 44.1 | 0.67 | 0.57 |
| Coughed | 27 | 12.3 | 2.3 | 22.4 | 35 | 20.0 | 8.1 | 31.9 | 0.20 | 0.26 |
| Felt ill | 27 | 9.9 | 1.6 | 18.2 | 35 | 8.6 | 0.0 | 17.2 | 0.77 | 0.47 |
| Pain killers | 27 | 33.3 | 7.6 | 59.0 | 35 | 48.6 | 25.2 | 72.0 | 0.23 | 0.23 |
| Nutritional supplements | 27 | 18.5 | -2.6 | 39.7 | 35 | 22.9 | 3.2 | 42.5 | 0.68 | 0.68 |
| Feeding tube | 27 | 0.0 | 0.0 | 0.0 | 35 | 0.0 | 0.0 | 0.0 |  |  |
| Weight loss | 27 | 14.8 | -4.5 | 34.2 | 35 | 17.1 | -0.5 | 34.8 | 0.81 | 0.81 |
| Weight gain | 27 | 37.0 | 10.7 | 63.4 | 35 | 34.3 | 12.1 | 56.5 | 0.83 | 0.82 |

**Web appendix 2 Figure WA1: Kaplan-Meier plots of time to recurrence (A) and overall survival (B) by randomised treatment group**

**A**

**12 TTR events/56 (21.4%)**

**9 TTR events/54 (16.7%)**

**Log-rank test p=0.75**

**HR (for CS-IMRT compared with 3DCRT : 1.16 (95% CI 0.48 – 2.79)**

**B**

**10 OS events/56 (17.9%)**

**11 OS events/54 (20.4%)**

**Log-rank test p=0.72**

**HR (for CS-IMRT compared with 3DCRT : 0.85 (95% CI 0.36 – 2.01)**
