# Supplementary material for: Results of a multicentre randomised controlled trial of cochlear-sparing intensity-modulated radiotherapy versus conventional radiotherapy in patients with parotid cancer (COSTAR; CRUK/08/004)
Source: Eur J Cancer. 2018 Nov;103:249–58. doi: 10.1016/j.ejca.2018.08.006 (PMC6202674; doi:10.1016/j.ejca.2018.08.006)
Supplement: Multimedia component 2 [file mmc2.pdf]

COSTAR

Target Volume Definition Guidelines

INTRODUCTION

Accurate target volume definition is an absolute requirement of radiotherapy planning. 3-Dimensional Conformal Radiotherapy (3D-CRT) and, in particular, Intensity Modulated Radiotherapy (IMRT), require detailed knowledge of CT-based anatomy. IMRT allows the delivery of very precise dose distributions, so that areas not specifically included in the target volume will not be treated to a therapeutic dose. Therefore, great care must be taken to ensure all the involved areas and those at risk are included.

The application of 3D-CRT and IMRT to head and neck cancers has resulted in standardised guidelines for the CT delineation of clinical target volumes (CTV) in the node negative neck, node positive neck and postoperative neck [1] . Clinical target volume definition of tumours of hypopharynx and oropharynx have also been proposed in the guidelines for the PARSPORT Trial[2].

This document describes target volume definition of the parotid bed and indications for ipsilateral neck irradiation for patients entering the COSTAR trial.

ANATOMY OF THE PAROTID GLAND

The parotid gland is the largest salivary gland. It measures approximately 6cm x 3.5cm. It is an irregular wedge shaped unilobular organ with autonomic innervation and encapsulating the facial nerve.

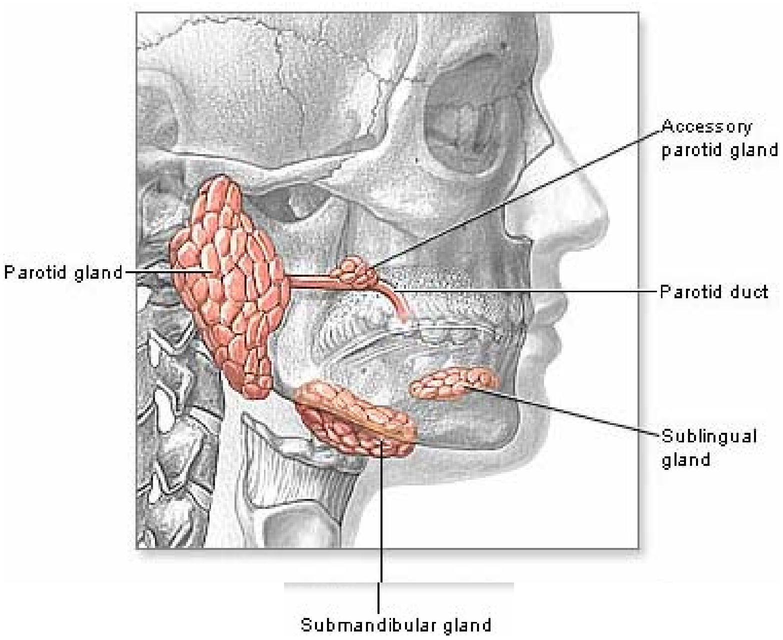

Figure 1: Major salivary glands

Anatomical borders

- tragus
- midpoint masseter
- angle of mandible
- mastoid process

It has five compartments: 3 superficial and 2 deep, all within a capsule in continuum with the deep cervical fascia. Stensen's duct is the parotid duct which lies anterior and exits the buccal mucosa at the level of the second upper molar.

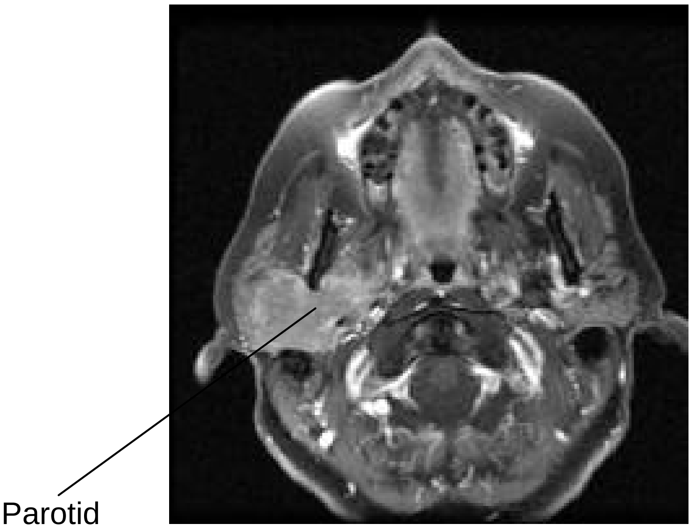

Figure 2: MRI axial image of right parotid tumour

The parotid gland lies in the parotid compartment which is contained within the following anatomical landmarks:

- Superior- zygomatic arch
- Inferior- styloid process
- Anterior- anterior border of masseter
- Posterior- mastoid process

Pathology

Malignant parotid tumours are commonly classified as low grade and high grade. Histological subtypes include: mucoepidermoid, acinic cell, adenocarcinoma, adenoid cystic carcinoma and squamous cell carcinoma.

Metastatic squamous cell carcinoma to the parotid and pleomorphic salivary adenomas are excluded from the COSTAR trial

Indications for postoperative radiotherapy to the parotid bed

These may include T3/T4 disease and or any of the following [3-8]:

- incomplete or close resection margins
- high grade histology
- recurrent disease
- peri-neural disease
- nodal involvement

Defining the parotid bed [9]

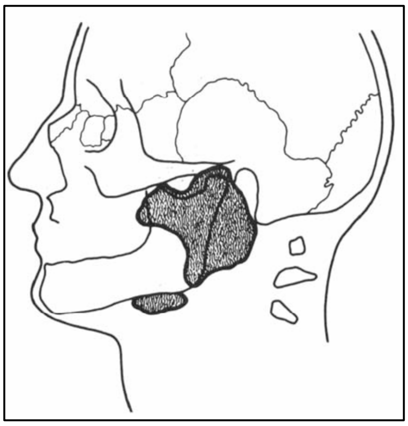

Figure 3: Sagittal representation

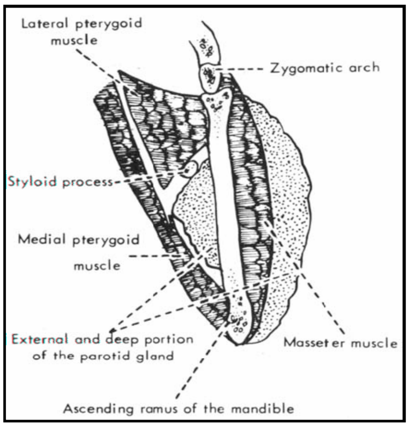

Figure 4: Coronal representation

- The parotid gland extends from the zygomatic arch superiorly to beyond the lower border of the mandible inferiorly (Fig.3).

- Posteriorly, the gland dips in the space between the mandible and the mastoid, with the adjoining external auditory meatus intimately surrounded by the gland in its free borders, that is, anteriorly and inferiorly.

- On a deeper plane, the parotid (Fig.4) is related to the styloid process and the muscles connected to it.

Locoregional involvement

Local infiltration of tissues adjacent to the parotid gland is the main pattern of spread. This follows the anatomical borders of the parotid gland and in cases of perineural invasion, the facial nerve to the stylomastoid foramina[3, 9].

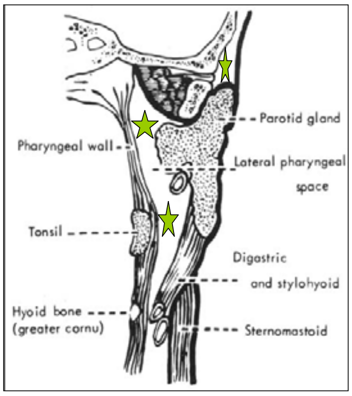

- Soft tissue extension-
  - The parotid gland, along with its associated periparotid lymph nodes, facial nerve and vessels lie within the parotid facial compartment
  - The fascia is deficient in the medial aspect, thus an increased risk of spread to the parapharyngeal space
- At risk areas:
  - Infratemporal fossa
  - Parapharyngeal space
  - Masseter
  - Diagastric
  - Skin

Figure 5: coronal image[9]

- Bone infiltration- at risk areas include:
  - Lateral part of the floor of middle cranial fossa
  - Neck of mandible
  - External auditory meatus
  - Inferior surface of styloid process

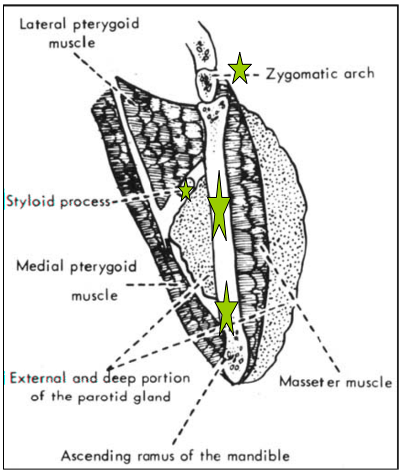

Figure 6: coronal image[9]

CLINICAL TARGET VOLUME DEFINITION

Following ICRU 50 and 62 guidelines [10, 11]

Clinical target volume (CTV):
It is a tissue volume that contains post-operative residual disease and/ or tissue at risk of harbouring microscopic disease or metastases. This volume then has to be treated adequately in order to achieve the aim of therapy: cure or palliation.

CTV1: clinical target volumes to be treated to a radical dose or post-operative dose

CTV2: clinical target volumes to be treated to an elective dose

CLINICAL TARGET VOLUME SELECTION

All treatment will be CT planned. The scans will be performed with the neck in extended position, scanning from the vertex to carina. CT scans will be taken at a maximum spacing of 3mm intervals through the primary tumour bed and the cervical lymph nodes.

The CTV will be constructed on each CT slice, as will critical structures. Diagnostic CT scans, MRI scans, clinical information, histopathology details and operative findings may be used to assist the definition of target volumes. Bilateral cochlea, spinal cord, brain stem, and eyes will be outlined.

CTV 1 includes:
- the parotid bed and any adjacent tissues at risk of microscopic spread
- Levels Ib, IIa IIb ipsilateral nodal stations

The TMG (Trial Management Group) recommends including ipsilateral lateral retropharyngeal, level Ib, IIa and IIb nodal stations within CTV1 as they are the first echelon nodes of parotid malignancies and are anatomically within or in close proximity to the parotid bed.

Please review the nodal guidelines attached for anatomical borders for Level Ib, IIa and IIb nodal stations.

CTV 2 includes:
- Levels III, IV and V ipsilateral nodal stations

OUTLINING THE PAROTID BED and FIRST ECHELON NODES (CTV1)

The parotid bed is approximately triangular in shape when seen on an axial CT image. The 3D volume when integrated on all the slices is pyramidal. The contralateral parotid gland is used as guidance to construct the parotid bed (CTV1).

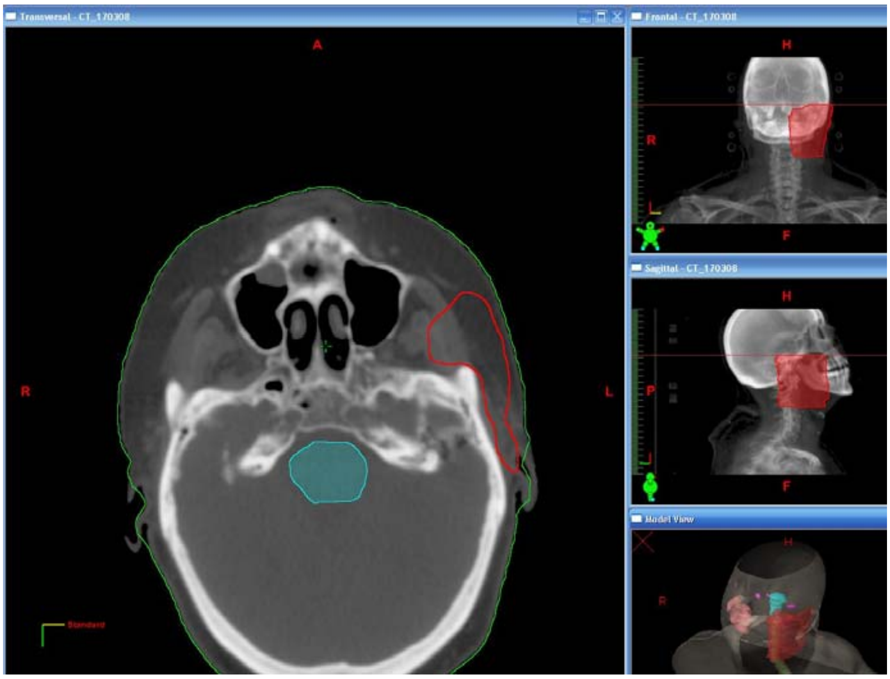

Figure 7: Superior-lateral extent of zygomatic arch

- **CT anatomical identification of superior border: superior-lateral extent of zygomatic arch**
  - o The CT slice passing through the most lateral and superior extent of the zygomatic arch (ie. above the temporomandibular joint). As represented in Figure 7, the posterior outline starts at the posterior attachment of the pinna and continues to the posterior part of the zygomatic arch following the soft tissue plane in front of the base of skull. Depending on the individual findings, medially the target volume continues into the infratemporal fossa, at least to the masseter. Anteriorly, the CTV includes the posterior half of the masseter. Lateral margin is the skin.

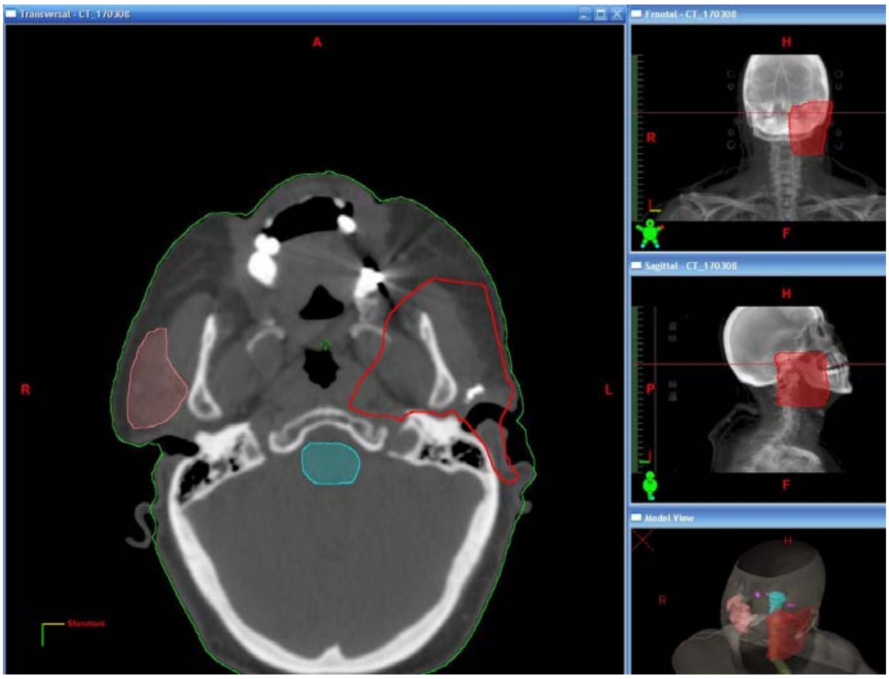

Figure 8: Level of external auditory canal

- **Above and at level of the external auditory canal**
  - o The anterior border follows the anterior border of the masseter then medially, the volume will include the parapharyngeal space and lateral pterygoid plate. Posteriorly, the CTV1 will follow the soft tissue plane to include the lateral retropharyngeal nodes and soft tissue anterior to the skull base. As the volume continues laterally, it includes external auditory canal and attachment of the pinna with the skin as the lateral border.

- **Below the level of the external auditory canal/ level of mastoid air cells (Fig: 9)**
  - o Once below the EAM the outline follows the posterior border of the mastoid process (the posterior extent of mastoid air cells) continuing to follow the soft tissue plane anterior to the skull base, including the lateral retropharyngeal nodes then continuing to include the parapharyngeal space. The anterior margin remains as the anterior border of the masseter and the lateral margin, the skin.

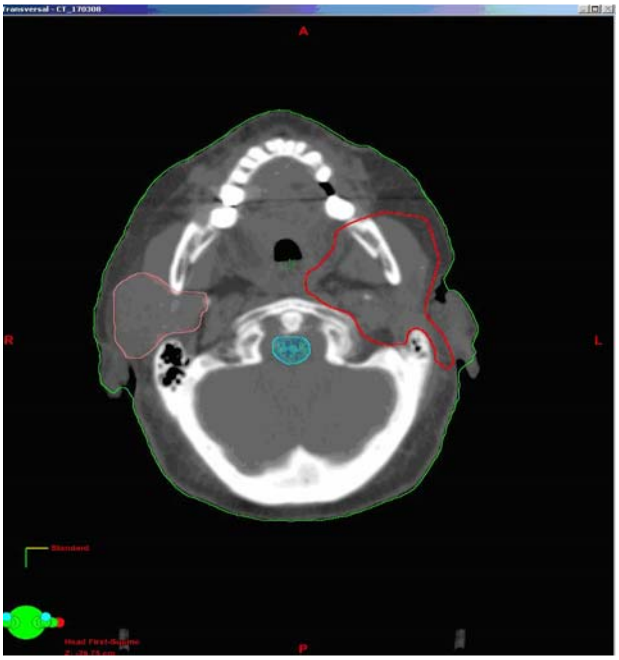

Figure 9: Mastoid air cells

- o Adenoid cystic carcinomas and in those with perineural invasion, the posterior margin must include the stylomastoid foramen.

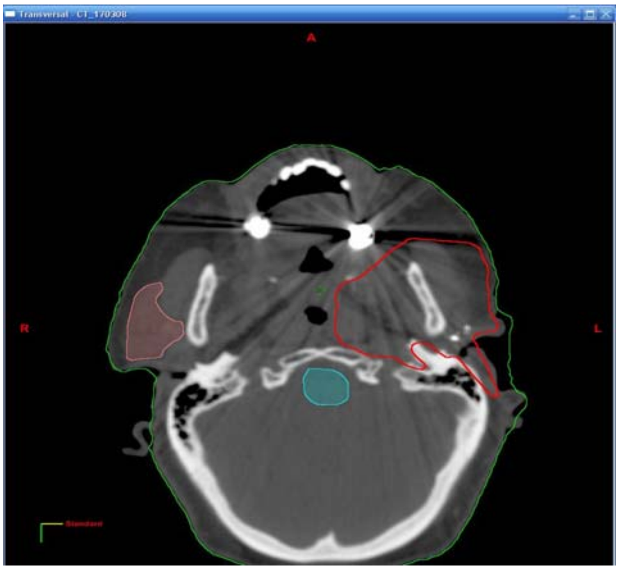

Figure 10: sternomastoid foramen

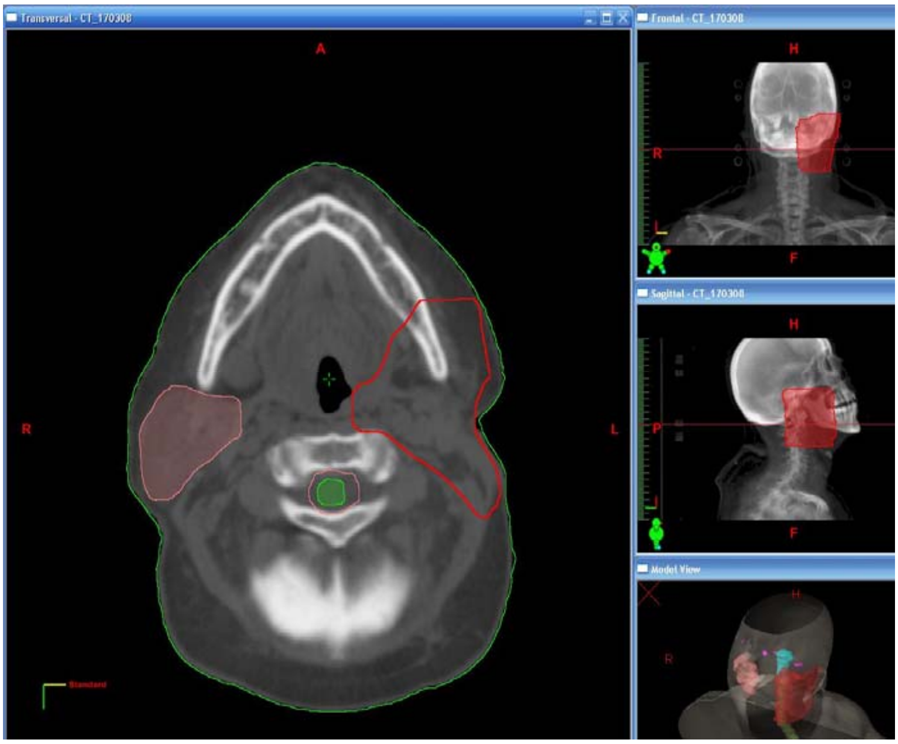

Figure 11: Angle of mandible

- **Level of angle of mandible**
  - o At this level, the anterior margin follows anterior to the submandibular gland and includes level Ib nodal volume. Medially, the margin will follow the medial border Level Ib and submandibular gland continuing to include the ipsilateral parapharyngeal space. The posterior margin includes Level IIa and IIb nodes and the lateral margins remain the same as described previously.

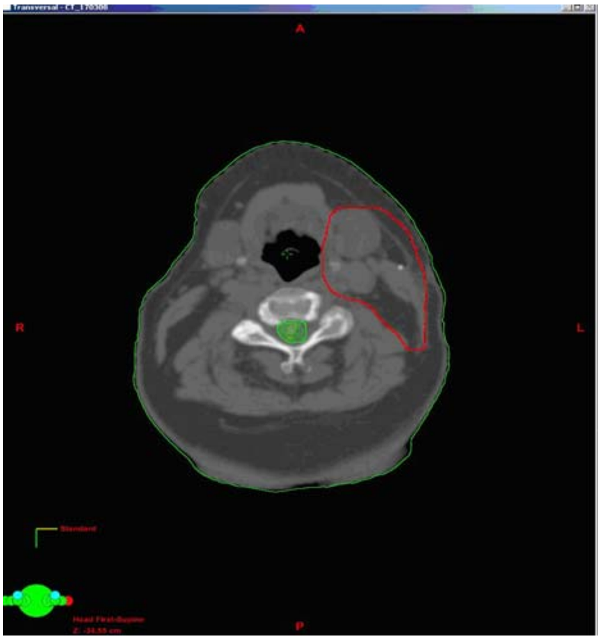

Figure 12: Superior edge of hyoid bone

- **Inferior level: superior cornu of the hyoid bone**

The lateral and medial outline continues as described above with the anterior margin to include the submandibular gland and the posterior margin to include the nodal outlining of level IIb (following back to the sternocleidomastoid muscle).

When to apply bolus to skin

Skin involvement has been demonstrated to be a poor prognostic indicator to developing distant metastases with a relatively low incidence for local recurrence despite using a skin sparing technique.

In order to obtain adequate skin dose, the TMG recommends application of bolus to the skin and or scar in the following situations:

- Skin involvement
- Close superficial margin
- Tumour spillage/ capsular rupture.

General points

- The margins described above may be adapted to include any bone infiltration.
- The mandibular ramus is included in the clinical target volume.
- Any air cavity is excluded from the clinical target volume.

Planning target volume

Planning Target Volume (PTV):
It is a geometric concept and is defined to select appropriate beam sizes and beam arrangements, taking into consideration the net effect of all the possible geometrical variations and inaccuracies in order to ensure that the prescribed dose is delivered to the CTV.

> PTV1: planning target volumes to be treated to a radical or post-operative dose
> PTV2: planning target volumes to be treated to an elective dose.

A margin will be added to the CTV, in all directions, to produce the PTV, in line with ICRU62 recommendations[10, 11].The magnitude of this margin will vary amongst different institutions and should reflect the geometric accuracy of their immobilisation system. This may vary between institutions from 3-5mm[12].

Departmental assessments of immobilisation systems may be required to define the margin.

THE ROLE OF NODAL IRRADIATION (CTV2)

The indications for elective irradiation of the neck in this cohort of patients are undefined. However, the presence of regional metastases has an important impact on the overall prognosis. Five-year survival rates can vary from 10% in node negative patients to 75% in node positive patients[13-15].

A review of the literature to date has detailed occult metastases arising in 12% to 48% of cases with N0 elective neck dissections reporting metastases in 20-50%[14, 16].

Single institution retrospective reviews have attempted to predict risk of nodal metastases[15].

- Univariate analysis: histological subtype, T stage, presence of desmoplasia, facial palsy, perineual invasion and extra-parotid extension.

- Mulitvariate analysis:
    - T stage (T3/T4),
    - desmoplastic reaction
    - histological subtype (adenocarcinaoma, undifferentiated carcinoma, high grade mucoepidermoid

Lymphatic drainage of the parotid include mostly subfacial and intraparotid nodes which are usually removed or within parotid bed. First echelon nodes are commonly Level IIa, alternatively, the parotid may drain directly to lateral retropharyngeal node, Level Ib nodes and terminating at Level III[13].

Elective neck irradiation

The following provides TMG consensus on the recommendations for elective nodal irradiation. Consider in any of the following:

- High grade tumours
- T3/T4
- Histological subtype
    - o Squamous cell carcinomas
    - o Adenocarcinomas
    - o Undifferentiated carcinomas,
    - o High grade mucoepidermoid carcinomas
    - o Salivary duct carcinomas

At least Level Ib-IV should be outlined

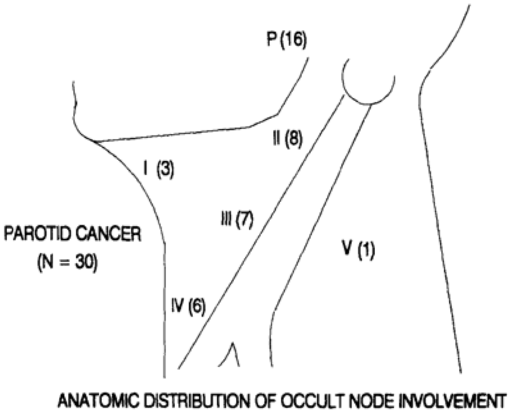

Figure 13: Occult nodal involvement of parotid malignancies[13]

Postoperative neck irradiation

The consensus guidelines suggest the following at risk groups that may benefit from postoperative radiotherapy:

- Multiple nodes
- >2 node levels involved
- ECS (extra capsular spread)
- N3 node (even if no detectable ECS)

Nodal outlining guidelines are attached to this document as Appendix 1.

OUTLINING ORGANS AT RISK

Organs at risk (OAR):

- Both cochlea
- Contralateral parotid gland
- Spinal cord (below foramen magnum)
- Brain stem (above foramen magnum)
- Lens

The following OARs should be outlined: both cochlea, the spinal cord, brain stem, eyes, contralateral parotid gland. A 3-5mm margin is added to spinal cord, brain stem. The cochlea PRV (planning risk volume) is outlined as described below.

Brain stem and spinal cord

The brain stem begins at the level of the foramen magnum, outlining of this structure must extend sufficiently superior to continue beyond the limit of irradiating fields.
.

The spinal cord begins below the level of the foramen magnum and extends inferiorly to the manubrium. The spinal cord is outlined, not the spinal canal.

OUTLINING THE COCHLEA

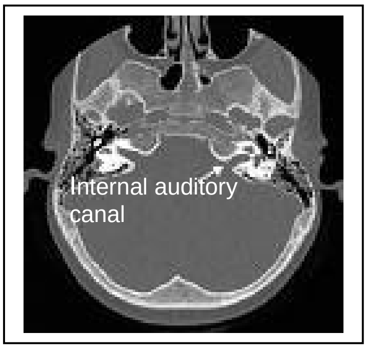

Figure 14: Axial image: internal auditory canal

The cochlea is a small structure present within the petrous temporal bone with an average volume of 0.56cm^3. The critical landmark in identifying the cochlea is the internal auditory canal. The cochlea is visualised superior to the lateral most end of the internal auditory canal. The vestibular apparatus can be visualised superior to the internal auditory canal. The cochlea is usually visible on up to three images when CT scans are performed at 3 mm intervals[17].

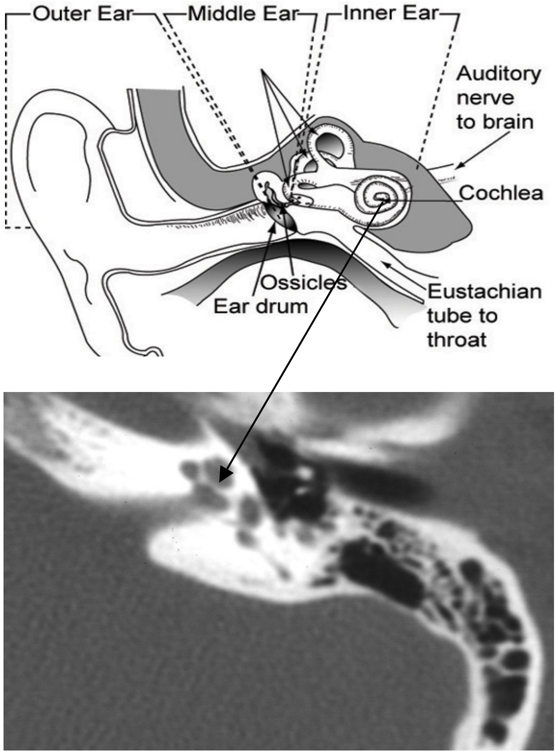

Figure 15 (a and b): Defining the vestibular apparatus

The cochlea is constructed by changing the Hounsfield Units (HUs) to achieve bone window settings. It is delineated on at least one CT axial image

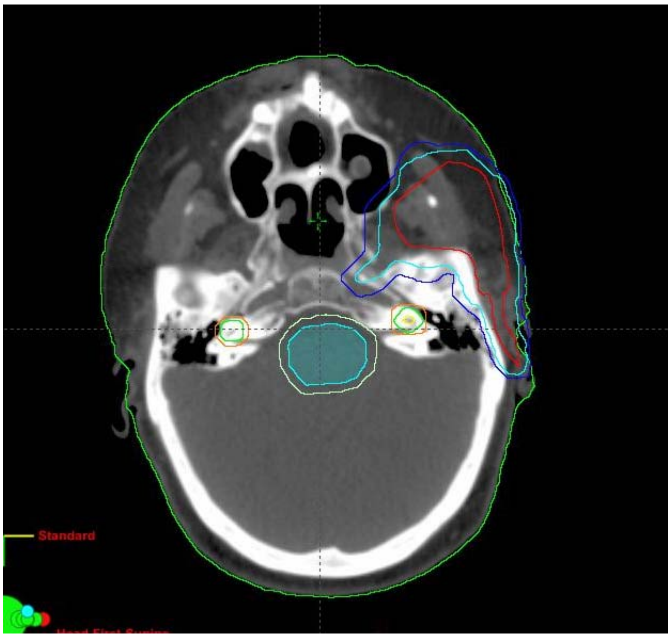

Figure 16: Axial image at level of auditory canal
Cochlea –yellow, PRV +3mm green, PRV +5mm orange

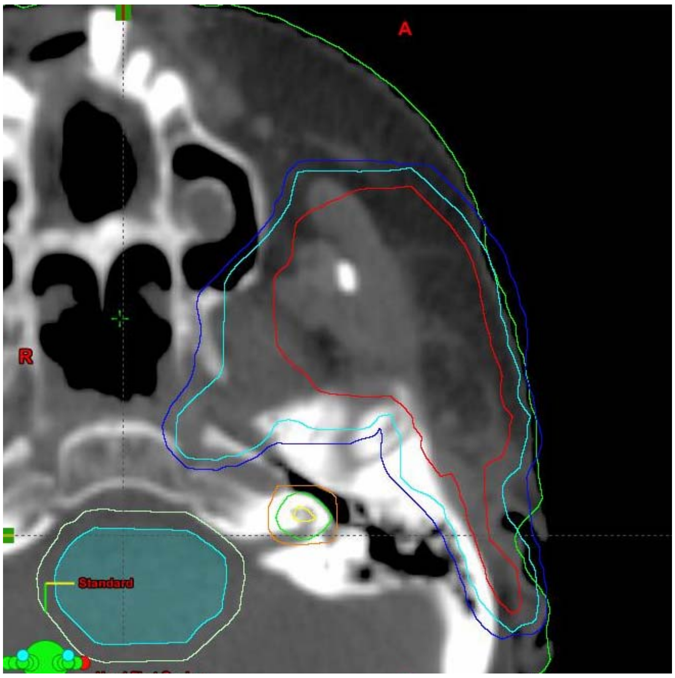

Figure 17: Axial image left cochlea (yellow) PRV 3mm green. CTV1 outlined in red and PTV (3mm) cyan and PTV (5mm) constructed in blue

The cochlea is too small to contour accurately. The TMG suggests attempting to outline the cochlea and add a 3mm margin circumferentially and longitudinally. This will create a PRV cochlea which is defined as the organ at risk. If it is difficult to outline the cochlea then the PRV cochlea may be outlined freehand. This can be constructed with the posterior wall of the external auditory canal anteriorly and including half the length of the internal auditory canal posteriorly (see Fig 17). Laterally, to include the internal auditory canal and thus complete a 7mm circle to the medial border. The TMG recommends 3mm PRV to be sufficient as literature review suggests immobilisation in the base of skull region to be reproducible to 2-3mm. However, some institutions may still wish to construct a 5mm margin.

The trial management group has made the following recommendations:

1. Ipsilateral lymph node groups Ib-V are contoured and defined as the 'at risk' nodal groups.
2. Ib, IIa and IIb are commonly within the surgical bed and hence will receive a dose of 60Gy in 30# and be incorporated into CTV1.
3. Patients who have not undergone neck dissection, lymph node groups III, IV and V are incorporated into CTV2 and will receive a dose of 50Gy in 25# (or 54Gy/30# IMRT arm) in the following groups:
    a. T3/T4
    b. All high grade tumours
    c. Histological subtypes:
        i. Squamous cell carcinomas
        ii. Adenocarcinomas
        iii. Undifferentiated carcinomas,
        iv. High grade mucoepidermoid carcinomas
        v. Salivary duct carcinomas
4. Post operative nodal irradiation is recommended in the following groups
    a. Extracapsular spread
    b. N2b disease
    c. N3

APPENDIX 1: OUTLINING NECK NODES
Target volume definition guidelines-PARSPORT Trial

Standardised guidelines for the CT delineation of clinical target volumes (CTV) in the node negative neck, node positive neck and postoperative neck have been published [1, 2, 18]

The following is an adjunct in outlining specific nodal groups.

NORMAL NODAL ANATOMY

Rouviere described in 1938 the anatomic details of the cervical lymphatic network[19]. He defined the first echelon lymph regions as those that receive drainage from certain organs. This is important when considering tumour spread. Also, although tumour spread along lymph vessels will be in the direction of drainage, in cases where there is a dilatation of the vessels due to an obstruction, normal circulation is arrested and retrograde spread may occur.

Figure 1: different nodal levels as described by Robbins[20]

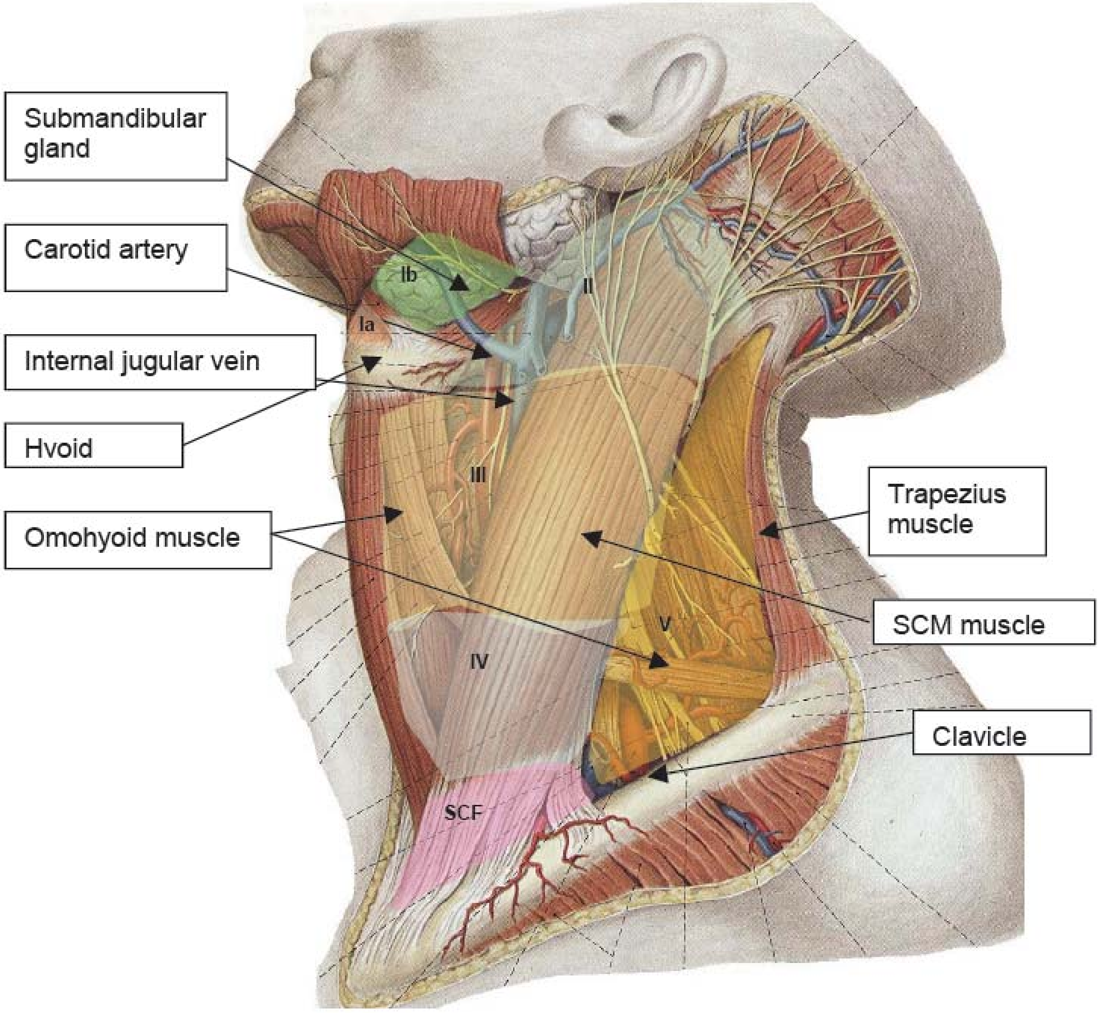

Sobotta 1982[21]

1. SUBMENTAL NODES (level Ia).

They are located in the submental region (figure 2) [21], which is bound:

- anteriorly by the mandible,
- posteriorly by the hyoid,
- laterally by the anterior bellies of the digastric muscles,
- superiorly by the muscles of the floor of the mouth, and
- inferiorly by the skin and subcutaneous tissue.

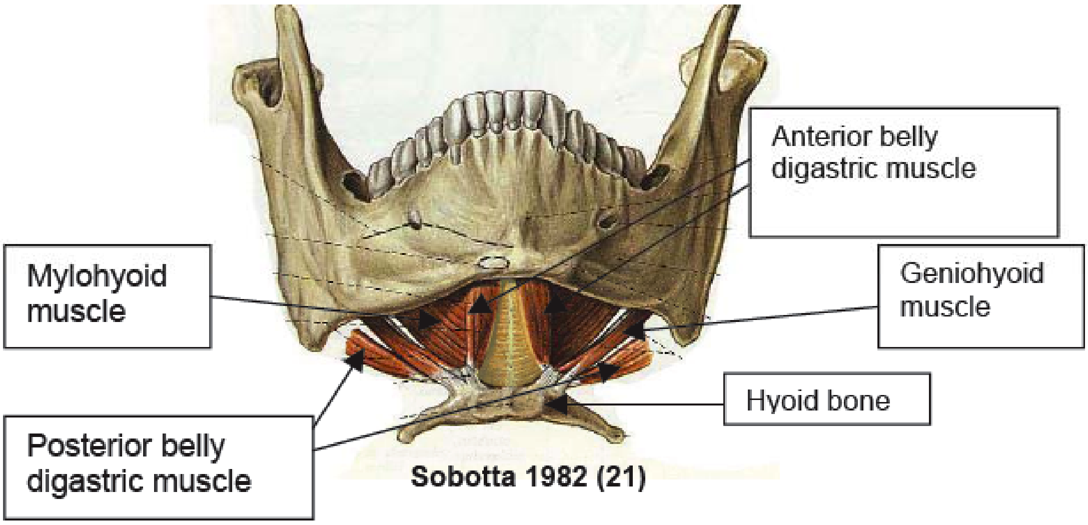

Figure 2: anatomical boundaries of the submental nodes

Level Ia	Anatomical boundaries					
Consensus Guidelines (4)	Cranial	Caudal	Anterior	Posterior	Lateral	Medial
	Geniohyoid, plane tangent to basilar edge of the mandible	Plane tangent to body of hyoid bone	Symphysis menti, platysma	Body of hyoid bone	Medial edge of anterior belly of digastric m	N/A

Notes:

1. Cranial margin: The geniohyoid muscle is one of the floor of mouth muscles, originating in mandible and inserting on the hyoid body.
2. Anterior margin: Follow the inner side of the mandible and, elsewhere, the platysma muscle. NOTE: **THE SKIN IS NOT PART OF THE NODAL CTV**, except where involved.
3. Posterior margin: There is small anatomical difference in patients treated with an extended neck and those with a neutral neck:
   i. Neutral neck: The chin is down and at the same level as the hyoid. The posterior margin in this case is the body of the hyoid bone.

ii. Extended neck: The chin is up and at higher level than the hyoid bone. In this case, on the inferior slices the posterior border remains the hyoid bone. At a more superior level it will correspond to the geniohyoid muscle.

4. Lateral margin: The medial edge of the anterior belly of the digastric muscle can sometimes be a little difficult to identify on CT. In these cases a line that goes from the symphysis mandible to the medial border of the parotid gland defines approximately the same space (as defined by Nowak et al [22])

2. SUBMANDIBULAR NODES (level Ib)

This space contains the submandibular gland and nodes. Its boundaries are usually easily identifiable on CT:

- anteriorly: body of the mandible,
- posteriorly: posterior edge of the submandibular gland,
- medially: anterior belly of the digastric muscle,
- laterally: mandible, platysma, and skin,
- superiorly: mylohyoid muscle,
- inferiorly: hyoid bone.

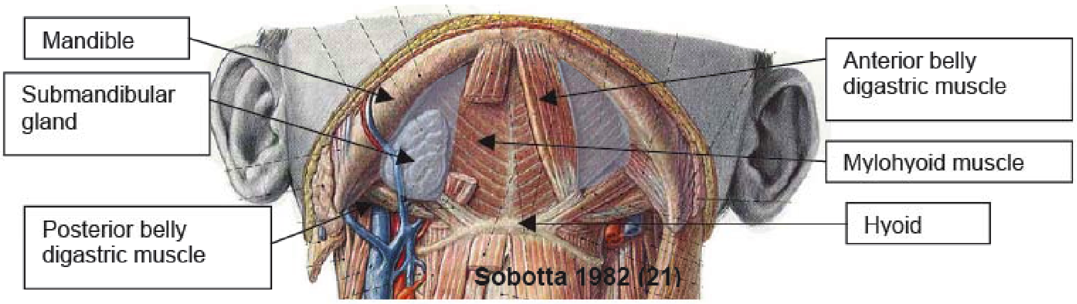

Figure 3: Anatomical landmarks- level Ib

Level IB	Anatomical boundaries					
Consensus guidelines (4)	Cranial	Caudal	Anterior	Posterior	Lateral	Medial
	Mylohyoid m., cranial edge of submandibular gland	Plane through central part of hyoid bone	Symphysis menti, platysma	Posterior edge of submandibular gland	Basilar edge/ inner side of mandible, platysma m., skin	Lateral edge of anterior belly of digastric muscle

The submandibular gland is included within this CTV. Some patients have large submandibular glands that extend more inferiorly than the proposed caudal boundary. In these cases we suggest that the entire gland is encompassed.

3. UPPER INTERNAL JUGULAR CHAIN (levels II)

Defined by Robbins as those nodes located around the upper third of the Internal Jugular Vein (IJV) and adjacent Spinal Accessory Nerve (SAN). They extend from the level of the Internal Carotid Artery (ICA) or hyoid bone to the skull base. The posterior boundary is the posterior border of the sternocleidomastoid (SCM) muscle and the anterior boundary is the lateral border of the sternohyoid muscle.

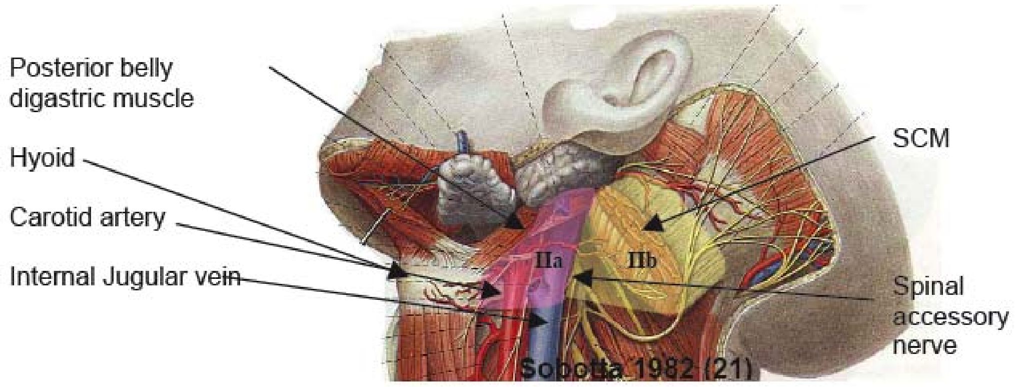

Figure 4: Anatomical landmarks- Level II

Level II can be subdivided into two sub-zones:

a. IIa are nodes located anterior to the SAN.
b. IIb located posterior to the nerve.

These two sub-zones represent 2 different groups involved in different patterns of lymphatic spread, with level IIb more likely to be associated with lesions of the

oropharynx and nasopharynx [23, 24]

Level II	Anatomical boundaries					
Consensus guidelines	Cranial	Caudal	Anterior	Posterior	Lateral	Medial
	Caudal edge of the lateral process of C1	Caudal edge of the body of the hyoid bone	IIa: Posterior edge of the sub-mandibular gland, anterior edge of ICA, posterior edge of posterior belly of digastric muscle.<br>IIb: Post border of internal jugular vein.	IIa: Post border of internal jugular vein IIb: posterior border SCM m.	Medial edge of SCM	Medial edge of ICA, paraspinal (levator scapulae) m.

The current proposed level, at the inferior edge of the lateral process of C1 falls a little short of the classical anatomical boundary. There is no evidence of increased recurrence in the spared tissue above it and it seems an appropriate boundary in the node negative neck and where there is no specific clinical indication to include the jugular fossa. Where indicated, extension of this boundary to the base of skull should be considered.

4. MID INTERNAL JUGULAR NODES (level III)

Defined as those nodes located around the middle third of the IJV, extending from the carotid bifurcation superiorly to the omohyoid muscle (or cricothyroid notch) inferiorly. The posterior boundary is the posterior border of the sternocleidomastoid muscle and the anterior boundary is the lateral border of the sternohyoid muscle.

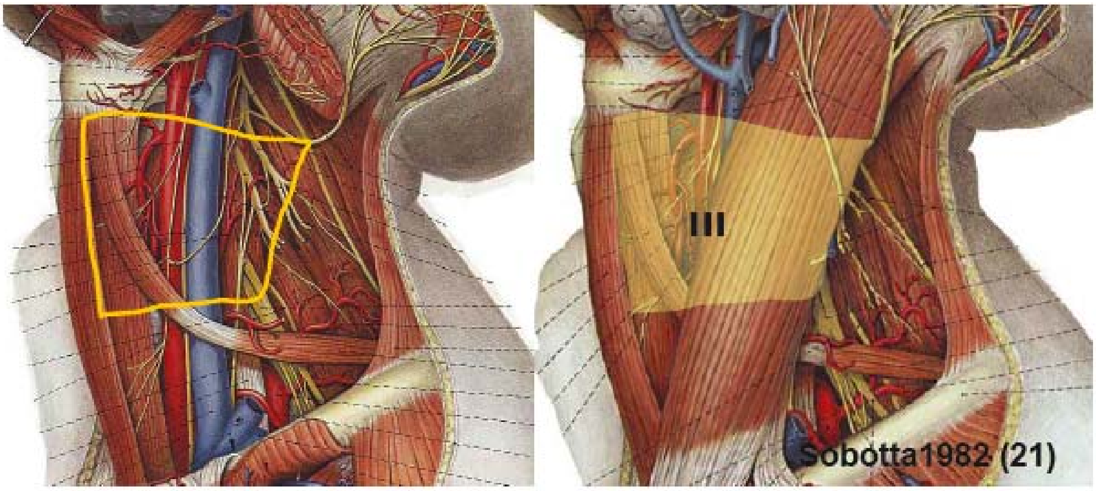

Figure 5: Anatomical landmarks- Level III

Level III	Anatomical boundaries					
Consensus guidelines	Cranial	Caudal	Anterior	Posterior	Lateral	Medial
	Caudal edge of the body of the hyoid bone	Caudal edge of the cricoid cartilage	Postero-lateral edge of the sternohyoid muscle, anterior edge of the SCM m.	Posterior edge of the SCM muscle	Medial edge of the SCM muscle	Internal edge of carotid artery, paraspinal (scalenius) muscle

5. LOWER INTERNAL JUGULAR CHAIN (level IV)

These are the nodes around the lower third of the IJV, extending from the omohyoid muscle superiorly to the clavicle inferiorly. The posterior boundary is the posterior border of the sternocleidomastoid muscle and the anterior boundary is the lateral border of the sternohyoid muscle.

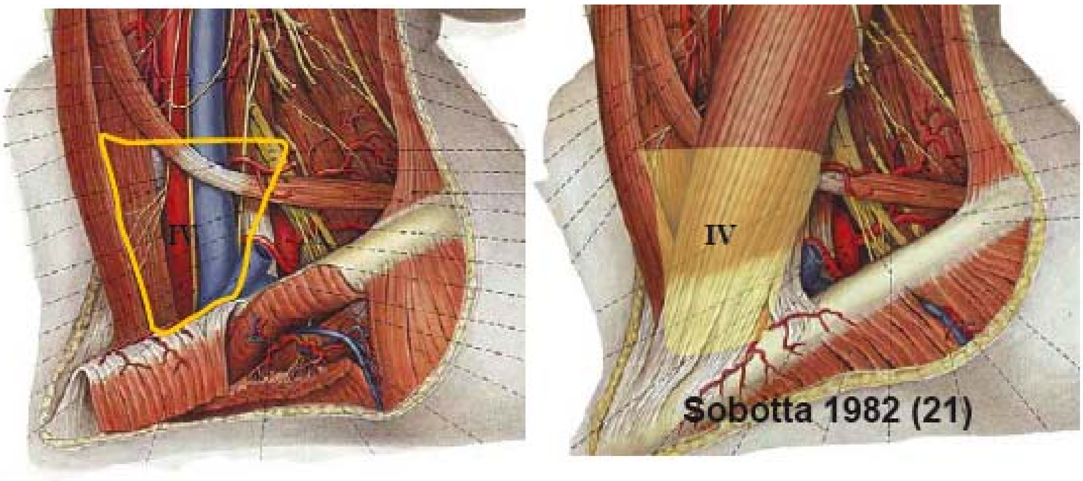

Figure 6: Anatomical landmarks- Level IV

Level IV	Anatomical boundaries					
Consensus Guidelines	Cranial	Caudal	Anterior	Posterior	Lateral	Medial
	Caudal edge of cricoid cartilage	2cm cranial to sternoclavicular joint	Antero-medial edge of SCM muscle	Posterior edge of SCM m	Medial edge of SCM m	Medial edge of ICA, paraspinal (scalenius) m.

The caudal edge of this level has been placed 2cm above the sterno-clavicular joint. Gregoire et al (2003) report that few surgical dissections extend down to the clavicle and that definitely they do not reach the medial portion of the clavicle at the level of the sterno-clavicular joint [18]. However there may be some local variation in surgical practice, and in some centres neck dissections do extend down to the level of the clavicle. Som placed the caudal end of both levels IV and V at the level of the top of the clavicle, as seen on each axial slice, considering nodes at or below the clavicle as supraclavicular nodes[25].

In most centres in the UK the inferior border of an anterior neck radiotherapy field is at the lower end of the medial aspect of the clavicle, with the lung apices shielded below the clavicle, and extending laterally to the coracoid process or to the junction of the medial 2/3 with the lateral 1/3 of the clavicle. Following the consensus guidelines would lead to a change in practice, which we do not advocate and therefore suggest the addition of a supraclavicular nodal level described below and not defined in the guidelines.

6. POSTERIOR TRIANGLE (level V)

Rouviere and Lindberg described the spinal accessory and transverse cervical chains [19, 26]

- Spinal accessory chain: group of nodes that blend with the IJC at its superior end and run along the spinal accessory nerve (SAN), extending from the superior portion of the sternocleidomastoid region, downward and outward, and following the nerve into the trapezius muscle where the chain terminates or more often continues to terminate along the superior border of the supra-spinous fossa. Where the nodes meet the trapezius muscle the chain merges with the transverse cervical chain.

- The transverse cervical chain extends transversely along the course of the transverse cervical artery, from the inferior extremity of the spinal accessory chain to the inferior extremity of the IJC (from the spinal accessory chain to the jugulo-subclavian junction).

Robbins defined the posterior triangle group or level V as the nodes located along the lower half of the spinal accessory nerve and the transverse cervical artery [20]. The boundaries for this nodal level were described as:

- Posteriorly: anterior border of the trapezius muscle,
- Anteriorly: posterior border of the sternocleidomastoid muscle
- Inferiorly: clavicle.

Level V was further sub-divided into 2 zones:

- Level Va: nodes along the lower part of the SAN
- Level Vb: nodes along the transverse cervical artery.

Rouviere and Som noted that the distinction between upper jugular nodes and upper spinal accessory nerve nodes might be impossible as these nodes merge at this level[20, 25, 27] Hamoir et al proposed a redefinition of the level Va boundaries [27],suggesting dividing it into:

- Vas, above the horizontal plane defined by the upper edge of the body of the hyoid. This area contains the superficial occipital group and contains few nodes, connected to the mastoid and skin. In 39 consecutive neck dissections, Hamoir et al found no LNs in this area in 32 cases, a single normal LN in 5 and 3 normal LNs in 2 cases.
- Vai: level V nodes between the upper edge of the body of the hyoid and the cricoid.

Robbins included the supraclavicular nodes in this group[20]. The UICC/AJCC 1997 [28]original description of this group (Ho's triangle) was those nodes in a

triangular plane defined by the upper sternal end of the clavicle, upper lateral end of the clavicle and the point at which the posterior portion of the neck meets the shoulder. Som placed the caudal end of both levels IV and V at the level of the clavicle, as seen on each axial slice, considering nodes at or below the clavicle as supraclavicular nodes[25]. The caudal portions of level IV and V can be within this triangle and it is often difficult to differentiate the different levels.

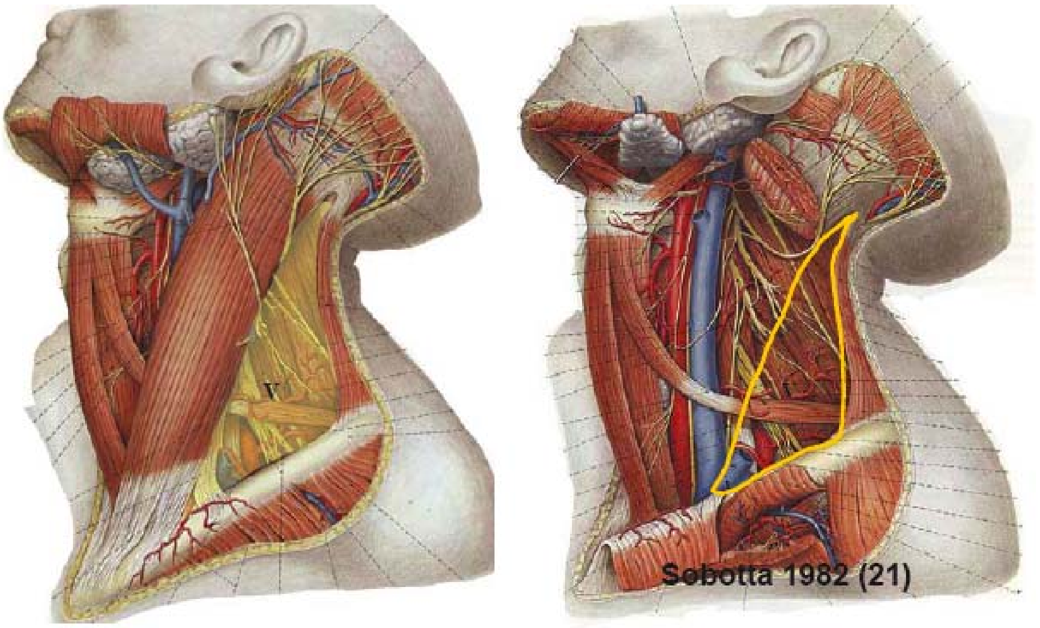

Figure 7: Anatomical landmarks- Level V

Level V	Anatomical boundaries					
Consensus guidelines	Cranial	Caudal	Anterior	Posterior	Lateral	Medial
	Cranial edge of body of hyoid bone	CT slice encompassing the transverse cervical vessels	Posterior edge of the SCM m	Antero-lateral border of the trapezius m	Platysma, skin	Paraspinal (levator scapulae, splenius capitis) m

The consensus guidelines have taken the cranial limit of level Vai nodes as proposed by Hamoir as the cranial edge of level V.
With regards to the caudal limit we have noted that sometimes the transverse cervical vessels are seen in more than one CT slice. We outline down to the lowest slice where these vessels can be seen.
In addition, below what has been defined as levels IV and V caudal limits, we add a supraclavicular volume, following the guidelines proposed by Som [25] as it is our policy with standard conventional radiotherapy to include the supraclavicular

nodes as part of level V. This level is in continuity with levels IV and V and encompasses the fatty planes and blood vessels at the root of the neck at the level of or just below the clavicle.

7. ANTERIOR NECK NODES (level VI)

Described by Rouviere [19] as the nodes situated below the hyoid and between the right and left carotid sheaths:

- Anterior Jugular chain: Follows the path of the anterior jugular vein. Situated in the space between the superficial cervical fascia and the sternocleidomastoid (SCM) muscle anteriorly and the pre-tracheal layer of the deep cervical fascia and the infra-hyoid muscles posteriorly. They receive afferents from the skin and muscles from the anterior part of the neck.
- Juxta-visceral nodes: pre-laryngeal, pre-thyroid, pre-tracheal and latero-tracheal, and the pre- tracheal and latero-tracheal nodes. They receive afferents from the thyroid gland, larynx, piriform fossae, trachea and oesophagus. They drain to the internal jugular chain.

Level VI	Anatomical boundaries					
	Cranial	Caudal	Anterior	Posterior	Lateral	Medial
Consensus guidelines	Caudal edge of body of the thyroid cartilage	Sternal manubrium	Skin, platysma	Separation between trachea and oesophagus	Medial edges of thyroid gland, skin and antero-medial edge of SCM m.	N/A

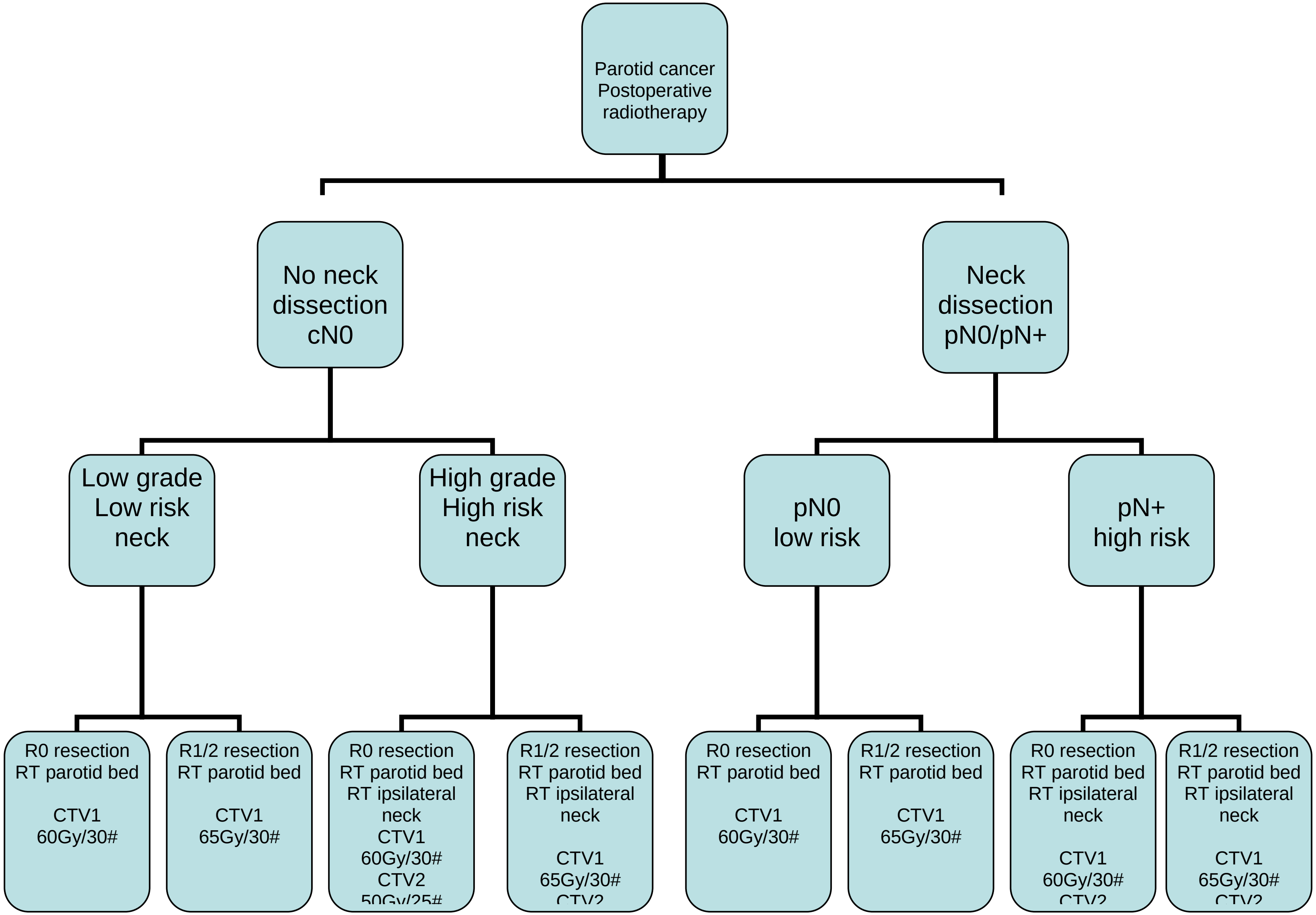
Parotid cancer Postoperative radiotherapy
No neck dissection cN0
Neck dissection pN0/pN+
Low grade Low risk neck
High grade High risk neck
pN0 low risk
pN+ high risk
R0 resection RT parotid bed CTV1 60Gy/30#
R1/2 resection RT parotid bed CTV1 65Gy/30#
R0 resection RT parotid bed RT ipsilateral neck CTV1 60Gy/30# CTV2
R1/2 resection RT parotid bed RT ipsilateral neck CTV1 65Gy/30#
R0 resection RT parotid bed CTV1 60Gy/30#
R1/2 resection RT parotid bed CTV1 65Gy/30#
R0 resection RT parotid bed RT ipsilateral neck CTV1 60Gy/30#
R1/2 resection RT parotid bed RT ipsilateral neck CTV1 65Gy/30#

REFERENCES

1. Gregoire, V., et al., *Selection and delineation of lymph node target volumes in head and neck conformal radiotherapy. Proposal for standardizing terminology and procedure based on the surgical experience.* Radiother Oncol, 2000. **56**(2): p. 135-50.
2. Guerrero Urbano, M.T., et al., *Target volume definition for head and neck intensity modulated radiotherapy: pre-clinical evaluation of PARSPORT trial guidelines.* Clin Oncol (R Coll Radiol), 2007. **19**(8): p. 604-13.
3. Chen, A.M., et al., *Local-regional recurrence after surgery without postoperative irradiation for carcinomas of the major salivary glands: implications for adjuvant therapy.* Int J Radiat Oncol Biol Phys, 2007. **67**(4): p. 982-7.
4. Bell, R.B., et al., *Management and outcome of patients with malignant salivary gland tumors.* J Oral Maxillofac Surg, 2005. **63**(7): p. 917-28.
5. Garden, A.S., et al., *Postoperative radiotherapy for malignant tumors of the parotid gland.* Int J Radiat Oncol Biol Phys, 1997. **37**(1): p. 79-85.
6. Pohar, S., et al., *Malignant parotid tumors: presentation, clinical/pathologic prognostic factors, and treatment outcomes.* Int J Radiat Oncol Biol Phys, 2005. **61**(1): p. 112-8.
7. Spiro, R.H., *Salivary neoplasms: overview of a 35-year experience with 2,807 patients.* Head Neck Surg, 1986. **8**(3): p. 177-84.
8. Terhaard, C.H., et al., *The role of radiotherapy in the treatment of malignant salivary gland tumors.* Int J Radiat Oncol Biol Phys, 2005. **61**(1): p. 103-11.
9. Beahrs, O.H., et al., *Surgical management of parotid lesions. Review of seven hundred sixty cases.* Arch Surg, 1960. **80**: p. 890-904.
10. *Prescribing, recording and reporting photon beam therapy ICRU report 50.* (1993), Bethesda: International Commission on Radiation Units and Measurement.
11. *Prescribing, recording and reporting photon beam therapy (Supplement to ICRU Report 50).* (1999), Bethesda: International Commission on Radiation Units and Measurement.
12. Humphreys, M., et al., *Assessment of a customised immobilisation system for head and neck IMRT using electronic portal imaging.* Radiother Oncol, 2005. **77**(1): p. 39-44.
13. Armstrong, J.G., et al., *The indications for elective treatment of the neck in cancer of the major salivary glands.* Cancer, 1992. **69**(3): p. 615-9.
14. Chen, A.M., et al., *Patterns of nodal relapse after surgery and postoperative radiation therapy for carcinomas of the major and minor salivary glands: what is the role of elective neck irradiation?* Int J Radiat Oncol Biol Phys, 2007. **67**(4): p. 988-94.
15. Regis De Brito Santos, I., et al., *Multivariate analysis of risk factors for neck metastases in surgically treated parotid carcinomas.* Arch Otolaryngol Head Neck Surg, 2001. **127**(1): p. 56-60.

16. Zbaren, P., et al., *Elective neck dissection versus observation in primary parotid carcinoma.* Otolaryngol Head Neck Surg, 2005. **132**(3): p. 387-91.
17. Pacholke, H.D., et al., *Contouring the middle and inner ear on radiotherapy planning scans.* Am J Clin Oncol, 2005. **28**(2): p. 143-7.
18. Gregoire, V., et al., *CT-based delineation of lymph node levels and related CTVs in the node-negative neck: DAHANCA, EORTC, GORTEC, NCIC,RTOG consensus guidelines.* Radiother Oncol, 2003. **69**(3): p. 227-36.
19. H.Rouviere, *Anatomy of the Human Lymphatic System.* 1938: Edwards Brothers, Inc.
20. Robbins, K.T., et al., *Standardizing neck dissection terminology. Official report of the Academy's Committee for Head and Neck Surgery and Oncology.* Arch Otolaryngol Head Neck Surg, 1991. **117**(6): p. 601-5.
21. Sobotta, P.P.a., *Atlas der Anatomie des Menschen*. 2000: Isevier GmbH, Urban & Fischer Verlag München.
22. Nowak, P.J., et al., *A three-dimensional CT-based target definition for elective irradiation of the neck.* Int J Radiat Oncol Biol Phys, 1999. **45**(1): p. 33-9.
23. Robbins, K.T., *Classification of neck dissection: current concepts and future considerations.* Otolaryngol Clin North Am, 1998. **31**(4): p. 639-55.
24. Dawson, L.A., et al., *Patterns of local-regional recurrence following parotid-sparing conformal and segmental intensity-modulated radiotherapy for head and neck cancer.* Int J Radiat Oncol Biol Phys, 2000. **46**(5): p. 1117-26.
25. Som, P.M., H.D. Curtin, and A.A. Mancuso, *An imaging-based classification for the cervical nodes designed as an adjunct to recent clinically based nodal classifications.* Arch Otolaryngol Head Neck Surg, 1999. **125**(4): p. 388-96.
26. Lindberg, R., *Distribution of cervical lymph node metastases from squamous cell carcinoma of the upper respiratory and digestive tracts.* Cancer, 1972. **29**(6): p. 1446-9.
27. Hamoir, M., et al., *A proposal for redefining the boundaries of level V in the neck: is dissection of the apex of level V necessary in mucosal squamous cell carcinoma of the head and neck?* Arch Otolaryngol Head Neck Surg, 2002. **128**(12): p. 1381-3.
28. AJCC, U., *TNM Atlas 4th Ed*. 1997, (Springer).
